# Light‐Activated In Situ Vaccine with Enhanced Cytotoxic T Lymphocyte Infiltration and Function for Potent Cancer Immunotherapy

**DOI:** 10.1002/advs.202403158

**Published:** 2024-07-02

**Authors:** Xian An, Zhuang Chen, Yi Luo, Peng Yang, Zuo Yang, Tiannan Ji, Yajing Chi, Shuyuan Wang, Ruili Zhang, Zhongliang Wang, Jianxiong Li

**Affiliations:** ^1^ Medical School of Chinese PLA & Department of Oncology Chinese PLA General Hospital Beijing 100193 P. R. China; ^2^ Lab of Molecular Imaging and Translational Medicine (MITM) Engineering Research Center of Molecular & Neuro‐imaging Ministry of Education School of Life Science and Technology Xidian University Xi'an Shaanxi 710126 P. R. China; ^3^ Department of Emergency The Fifth Medical Center of PLA General Hospital Beijing 100193 P. R. China; ^4^ School of Medicine Nankai University Tianjin 300071 P. R. China

**Keywords:** cGAS‐STING pathway, cytotoxic T lymphocytes, immunogenic cell death, in situ cancer vaccine, tumor microenvironment

## Abstract

In situ cancer vaccination is an attractive strategy that stimulates protective antitumor immunity. Cytotoxic T lymphocytes (CTLs) are major mediators of the adaptive immune defenses, with critical roles in antitumor immune response and establishing immune memory, and are consequently extremely important for in situ vaccines to generate systemic and lasting antitumor efficacy. However, the dense extracellular matrix and hypoxia in solid tumors severely impede the infiltration and function of CTLs, ultimately compromising the efficacy of in situ cancer vaccines. To address this issue, a robust in situ cancer vaccine, Au@MnO_2_ nanoparticles (AMOPs), based on a gold nanoparticle core coated with a manganese dioxide shell is developed. The AMOPs modulated the unfavorable tumor microenvironment (TME) to restore CTLs infiltration and function and efficiently induced immunogenic cell death. The Mn^2+^‐mediated stimulator of the interferon genes pathway can be activated to further augment the therapeutic efficacy of the AMOPs. Thus, the AMOPs vaccine successfully elicited long‐lasting antitumor immunity to considerably inhibit primary, recurrent, and metastatic tumors. This study not only highlights the importance of revitalizing CTLs efficacy against solid tumors but also makes progress toward overcoming TME barriers for sustained antitumor immunity.

## Graphical abstract

A robust in situ cancer vaccine, Au@MnO_2_ nanoparticles (AMOPs), is developed to enhance the function and infiltration of Cytotoxic T lymphocytes via overcoming tumor hypoxia and extracellular matrix barriers. The AMOPs can not only inhibit subcutaneous tumor growth but also establish strong immunological memory and effectively prevent tumor recurrence and metastasis.

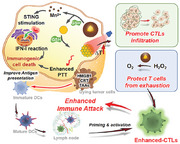

## 1 Introduction

In situ cancer vaccines use tumors as a natural reservoir of tumor antigens to stimulate the adaptive immune response against tumor cells, and this has gradually become an innovative strategy in immunotherapy.^[^ ^1^ ^]^ The antitumor efficacy of in situ cancer vaccines depends on cytotoxic T lymphocytes (CTLs) to infiltrate the primary tumor, a process considerably influenced by the quantity and quality of these immune cells.^[^ ^2^ ^]^ Studies have shown that memory T cells, which are crucial for sustained immune defense, are derived from CTLs that have escaped apoptosis.^[^ ^3^ ^]^ This highlights the importance of CTLs infiltration owing to the key role of CTLs tumor elimination and establishing long‐term immunological memory. Therefore, an effective in situ cancer vaccine must achieve two core objectives: prompting strong immunogenic cell death (ICD) at the tumor site and encouraging the infiltration and function of CTLs, thereby enhancing the outcome on primary tumors and promoting the creation of lasting immune memory.

Currently, most research has focused on efficiently inducing ICD with local therapy (such as photothermal therapy (PTT),^[^ ^4^ ^]^ photodynamic therapy,^[^ ^5^ ^]^ and radiation therapy^[^ ^6^ ^]^) and combining toll‐like receptor agonists^[^ ^7^ ^]^ or stimulator of interferon genes (STING) agonists^[^ ^8^ ^]^ to improve the antigenicity and adjuvanticity of the vaccine. While these methods are important for the priming of the immune response, several limitations remain in promoting the infiltration and function of CTLs in the tumor.^[^ ^9^ ^]^ In situ cancer vaccines can also be improved in this avenue because of these shortcomings. Although ICD induction and the simultaneous use of immune agonists are fundamental methods in vaccine design, the infiltration and function of CTLs in the tumor microenvironment (TME) are critical to ensure efficient vaccine performance. To maximize the potential of in situ cancer vaccines, the challenges arising from the TME must therefore be overcome. The dense extracellular matrix (ECM)^[^ ^10^ ^]^ and hypoxia conditions^[^ ^11^ ^]^ in the TME impede effective CTLs infiltration and functioning.^[^ ^12^ ^]^ For instance, the ECM acts as a physical barrier that hinders effective T‐cell migration and subsequent interactions. Meanwhile, hypoxic conditions decrease the survival and efficacy of CTLs, which then causes the failure of vaccine‐induced tumor clearance. Therefore, developing a novel in situ cancer vaccine is necessary to address these problems by promoting CTLs penetration via normalizing the ECM, improving tumor oxygenation to enhance CTLs function, and ensuring effective induction of ICD at the tumor site.

**Scheme 1:**
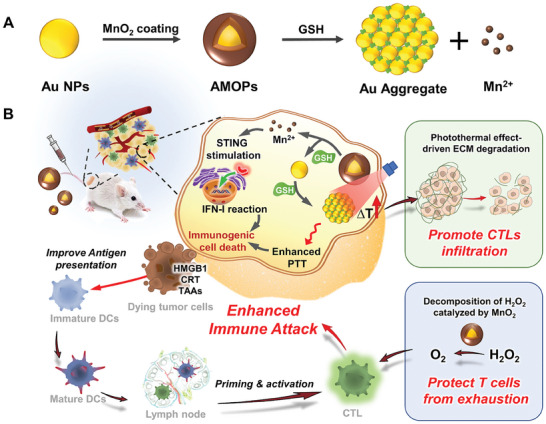
Schematic illustration of antitumor immunotherapy induced by laser: AMOPs vaccination strategy. A) Synthesis of AMOPs. B) AMOPs are intravenously injected and accumulated in tumors. Upon laser irradiation, they evoked immunogenic death of tumor cells, which release TAA and DAMPs to stimulate DC maturation. The presence of a dense ECM and hypoxic microenvironment in the tumor hinder the subsequent immune response. PTT‐induced ECM degradation and H_2_O_2_ are catalyzed to produce O_2_, enhancing CTLs infiltration and antitumor immunotherapeutic efficiency.

Herein, we propose a novel approach to develop a CTLs‐enhanced in situ cancer vaccine by combining the synergistic capabilities of two different functional materials: gold nanoparticles (Au NPs) and manganese dioxide (MnO_2_), termed AMOPs (**Scheme** **1**A). The MnO_2_ shell catalytically converts the overproduced hydrogen peroxide (H_2_O_2_) in the TME into oxygen (O_2_) and thus alleviates hypoxic conditions and augments the vitality and function of CTLs.^[^ ^13^ ^]^ Upon internalization by cancer cells, the MnO_2_ coating degrades because of the high levels of intracellular glutathione (GSH), resulting in the release of Mn^2+^. These Mn^2+^ ions serve as STING agonists and thereby amplify the adjuvant effect of the in situ cancer vaccines.^[^ ^14^ ^]^ Concurrently, the naked Au NPs are crosslinked by the excess GSH, shifting their absorption spectrum from the visible to the near‐infrared (NIR) region and activating the PTT ability.^[^ ^15^ ^]^ This PTT not only effectively induces ICD in tumor cells but also facilitates the degradation of ECM to promote the infiltration of CTLs. Thus, the AMOPs vaccine creates an environment that supports CTLs infiltration and function while efficiently inducing ICD (Scheme 1B). The efficacy of AMOPs‐driven photoactivated vaccine was demonstrated in a 4T1 mouse model, via its remarkable ability to inhibit the subcutaneous tumor, prolong survival, and establish immunological memory. Furthermore, the AMOPs vaccine induced an effective inhibition of secondary tumor growth and metastasis. This study reveals the critical role of CTLs in vaccine efficacy and provides new insights into the development of in situ cancer vaccines.

## 2 Results and Discussion

### 2.1 Preparation and Characterization of AMOPs

**Figure 1:**
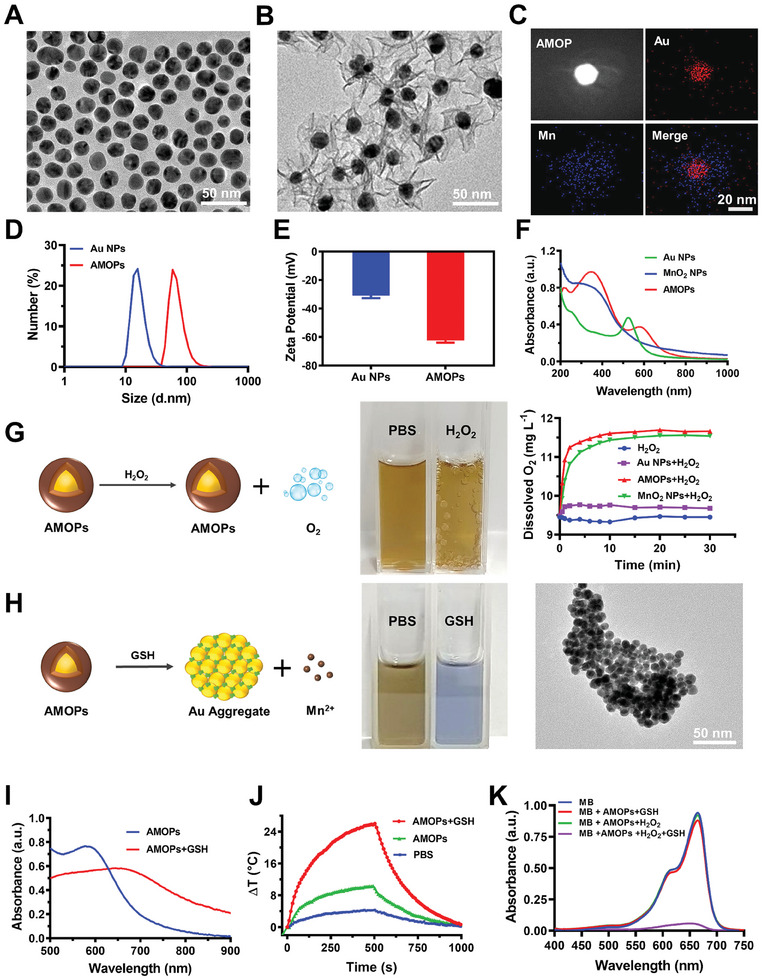
Characterization of AMOPs. Transmission electron microscopy images of A) Au NPs and B) AMOPs; scale bar: 50 nm. C) Energy dispersive spectroscopy elemental mapping of AMOPs obtained from; scale bar: 20 nm. D) Size and E) zeta potential of Au NPs and AMOPs. F) Absorption spectra of Au NPs, MnO_2_ NPs, and AMOPs. G) Characterization of AMOPs catalyzing hydrogen peroxide to generate O_2_. H) Characterization of responsive behavior of AMOPs in the presence of GSH. Note: the photographs in G and H were taken after reaction for 30 min with H_2_O_2_ or GSH. I) Absorption spectra and J) temperature change of AMOPs in PBS or GSH solution under 808 nm laser irradiation, with PBS as blank control. K) The degradation of methylene blue (MB), is used to represent •OH production.

To prepare AMOPs, Au NPs with a diameter of 15 nm were synthesized by reducing chloroauric acid with sodium citrate.^[^ ^16^ ^]^ Subsequently, MnO_2_ was coated onto Au NPs by reducing KMnO_4_ in situ, utilizing the citrate ligands on the surface. The sizes of Au NPs and AMOPs were then assessed using transmission electron microscopy (TEM) and dynamic light scattering (**Figure** **1**A,B,D). The AMOPs exhibited a consistent core–shell structure with an average diameter of ≈59 nm. To evaluate the composition of the core–shell nanostructure, an elemental map of the AMOPs obtained from energy dispersive spectroscopy demonstrated that the core contained Au (Figure 1C, red) and the shell contained Mn (Figure 1C, blue). With the successful coating of MnO_2_, the surface potential of AMOPs shifted from −30.9 to −62.4 mV (Figure 1E). The absorption spectra showed two characteristic absorption peaks of AMOPs at 350 and 580 nm, which could be assigned to the MnO_2_ absorbance peak and the red‐shifted peak of Au NPs in the formed AMOPs core–shell structure (Figure 1F).

MnO_2_ can catalyze the production of O_2_ from H_2_O_2_, a reaction that can mitigate tumor hypoxia and augment the efficacy of CTLs. Consequently, we explored the potential of AMOPs to serve as a catalyst for H_2_O_2_ decomposition. The results (Figure 1G; Figures S1 and S2, Supporting Information) confirmed that AMOPs retained the catalytic capabilities of MnO_2_, demonstrating good catalysis of H_2_O_2_ decomposition and generation of O_2_. This suggests that AMOPs can potentially improve oxygenation within the tumor region, thereby enhancing the activity and function of CTLs. We subsequently explored the performance of AMOPs in response to GSH (Figure 1H; Figure S3, Supporting Information). In the presence of GSH, the purple AMOPs solution immediately turned blue, indicating a fast response time. TEM imaging showed that the MnO_2_ shell had disappeared and that the Au NPs had assumed an aggregate morphology. The absorption of the AMOPs before and after interaction with GSH was analyzed through absorption spectroscopy. In the absence of GSH, the absorption peak of the AMOPs solution was observed at 578 nm and exhibited weak absorbance in the NIR region, similar to that of Au NPs (Figure 1I, blue line). However, the inclusion of GSH (illustrated by the red line in Figure 1I) markedly intensified the absorbance across the entire NIR spectrum, a phenomenon that correlates with the aggregation of Au NPs. This shift in absorption accordingly endows the AMOPs with GSH‐triggering photothermal characteristics (Figure 1J). Subsequently, our results illustrate that AMOPs showed good photothermal performance and photostability in the presence of GSH (Figure S4, Supporting Information). This activatable photothermal property imparts the ability of AMOPs to induce ICD in tumor cells while also allowing their efficient ECM clearance effects, providing a strong functional basis for in situ cancer vaccines. Additionally, the release of Mn^2+^ from the MnO_2_ shell triggers a Fenton‐like reaction that generates hydroxyl radicals (•OH) (Figure 1K; Figures S5–S7, Supporting Information). Thus, AMOPs can not only initiate ICD in cancer cells via PTT process but also inflict DNA damage through the CDT process. Such functions help create an environment that favors the activation of the STING pathway.

The biostability of the AMOPs was evaluated by measuring the hydrodynamic diameter, which was negligible after 48 h of incubation in phosphate‐buffered saline (PBS), culture medium, and serum (Figure S8, Supporting Information). Meanwhile, we verified that AMOPs at concentrations up to 200 µg mL^−1^ did not induce hemolysis (Figure S9, Supporting Information). Thus, AMOPs meet the need for stability and safety in subsequent cell and animal experiments.

### 2.2 Antitumor Effects of AMOPs‐Mediated Therapy In Vitro

**Figure 2:**
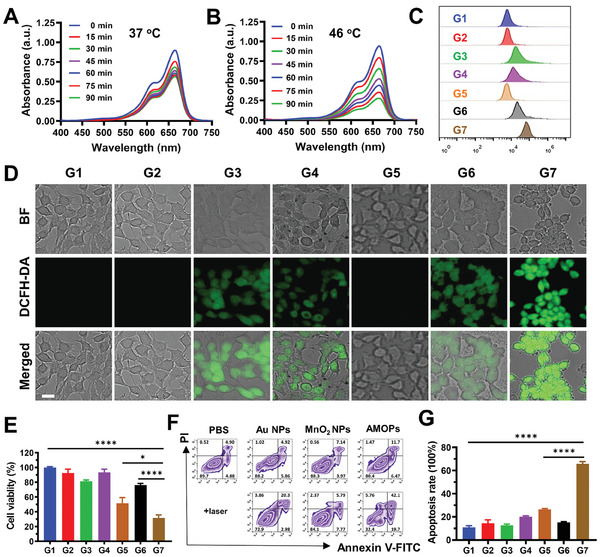
In vitro laser induction of antitumor effects of AMOPs. The degradation of MB by AMOP in solutions containing A) H_2_O_2_ and B) GSH at different times under conditions of 37 or 46 °C, respectively. C) ROS determined by flow cytometry of 4T1 cells in different treatment groups. D) Representative fluorescence images of cellular ROS production in 4T1 cells of different treatment groups. E) MTT assay of 4T1 cells with different treatments (*n* = 5). F) Flow cytometry analysis of 4T1 cells costained with Annexin V‐FITC and PI after different treatments and G) corresponding apoptosis rates. Data are presented as the mean ± SD. (*n* = 3). Groups are G1, PBS; G2, Au NPs; G3, MnO_2_ NPs; G4, AMOPs; G5, Au NPs + Laser; G6, MnO_2_ NPs + Laser; and G7, AMOPs + Laser. Data are presented as the mean ± SD. Significance was calculated via unpaired *t*‐test (^*^ *p* < 0.05; ^**^ *p* < 0.01; ^***^ *p* < 0.001; ^****^ *p* < 0.0001).

To verify the cellular uptake capacity, TEM images of 4T1 cells were characterized after incubation with different concentrations of AMOPs (Figure S10, Supporting Information). The results confirmed that AMOPs can indeed enter cells and transform into Au aggregates. Notably, as the concentration of AMOPs increased, so did the degree of aggregation, a concentration‐dependent photothermal effects were observed. Then, we further investigated the relationship between PTT and CDT mediated by AMOPs. The methylene blue degradation rate increased significantly with increasing temperature (**Figure** **2**A,B), highlighting the role of temperature as a key influencing factor in CDT performance. Subsequently, 4T1 cells were stained with 2′,7′‐dichlorodihydrofluorescein diacetate (DCFH‐DA) for the assessment of intracellular reactive oxygen species (ROS) levels across different experimental groups via flow cytometry and fluorescence imaging. Group G7 (AMOPs + laser), representing the CDT process enhanced by photothermal sensitization, achieved the highest fluorescence intensity compared with other groups (Figure 2C,D). The fluorescence intensities of groups G3 (MnO_2_), G4 (AMOPs), and G6 (MnO_2_+L) were similar and slightly lower than that of the G7 group. These three groups, which all contained Mn^2+^ and were not exposed to laser irradiation, represented simple CDT processes. Conversely, the fluorescence intensities for groups G2 (Au NPs) and G5 (Au NPs + laser) did not exhibit a significant enhancement in comparison with that of the control group G1 (PBS), indicating the necessity of Mn^2+^ for the increase in intracellular ROS. Thus, the CDT process was effectively enhanced by AMOPs‐mediated PTT, thereby improving the potential for cell killing.

We then investigated the effects of AMOPs‐mediated treatment under various conditions. At elevated concentrations (200 µg mL^−1^) in the absence of light, AMOPs exhibited acceptable toxicity in L‐02 and 4T1 cells (Figure S11, Supporting Information). Upon laser irradiation, as observed in the G5 and G7 groups, the cancer‐cell killing rate reached 48.7% and 68.2%, respectively (Figure 2E). The efficacy of laser‐activated AMOPs was further substantiated through FACS analysis of apoptosis and live/dead cell staining (Figure 2F,G; Figure S12, Supporting Information). Therefore, AMOPs were shown to be highly effective in killing cancer cells upon laser irradiation, leveraging the combination of PTT and PTT‐enhanced CDT.

### 2.3 AMOPs‐Mediated Therapy Induced ICD and Activated STING Pathway In Vitro

**Figure 3:**
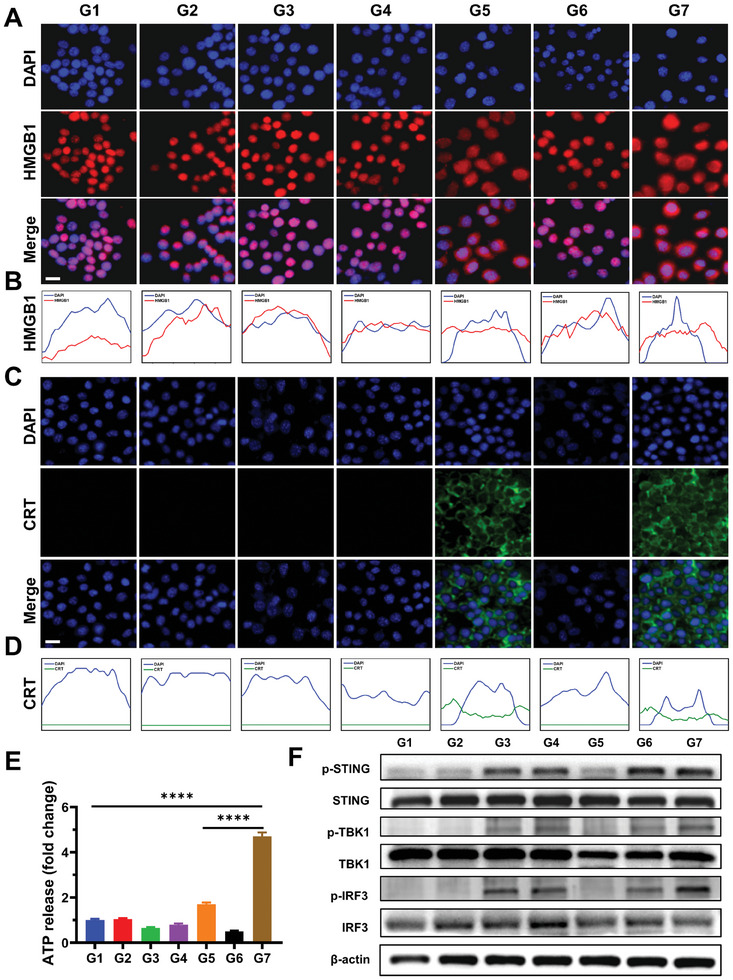
AMOPs‐mediated therapy induced ICD and activated STING pathway in vitro. A) Representative fluorescence images and B) Colocation maps of HMGB1 release in 4T1 cells after different treatments; scale bar: 20 µm. C) Representative fluorescence images and D) colocation maps of CRT exposure on the surface of 4T1 cells after different treatments; scale bar: 20 µm. E) Detection of ATP release in media from 4T1 cells after different treatments (*n* = 3). F) Western blot analysis of cGAS‐STING pathway activation in DC2.4 cells after treatment with indicated groups. Groups are G1, PBS; G2, Au NPs; G3, MnO_2_ NPs; G4, AMOPs; G5, Au NPs + Laser; G6, MnO_2_ NPs + Laser; and G7, AMOPs + Laser. Data are presented as the mean ± SD. Significance was calculated via unpaired *t*‐test (^****^ *p* < 0.0001).

Having demonstrated that AMOPs effectively kill cancer cells with laser irradiation, we then sought to address whether this cytotoxic effect induces ICD. The release of high‐mobility group box 1 (HMGB1) and the translocation of calreticulin (CRT) are key hallmarks of ICD, both enhancing the antigen uptake and presentation capabilities of antigen‐presenting cells.^[^ ^17^ ^]^ Consequently, detecting the release of HMGB1 and the translocation of CRT via immunofluorescence staining serves as an effective method for assessing ICD. HMGB1 was predominantly localized in the nuclei of cells from groups without laser irradiation, such as G2 (Au NPs), G3 (MnO_2_ NPs), and G4 (AMOPs) (**Figure** **3**A,B), with no significant difference from that in the control group G1 (PBS). Conversely, in groups under laser irradiation, G5 (Au NPs + Laser) and G7 (AMOPs + Laser), HMGB1 was translocated from the nucleus to the cytoplasm and was subsequently released because of cell death (Figure S13, Supporting Information). In group G6 (MnO_2_ + Laser), the HMGB1 fluorescence remained within the nucleus, suggesting that HMGB1 release was primarily attributed to the temperature elevation associated with PTT rather than the laser irradiation alone. Similarly, the CRT fluorescence signal was exclusively detected in groups G5 and G7, indicating that the AMOPs‐facilitated PTT process triggered ICD (Figure 3C,D). Moreover, ATP release, another critical hallmark of ICD, exhibits a positive correlation with cellular autophagy.^[^ ^18^ ^]^ The G7 (AMOPs + Laser) group demonstrated the synergistic impact of PTT and CDT, exposing cells to intense heat and oxidative stress, thereby eliciting the highest level of autophagy observed across the groups. As shown in Figure 3E, ATP release in the G7 group was significantly elevated compared with that in other groups and was 3.71‐fold higher than that of the control PBS group.

Mn^2+^ released from AMOPs within cells serves a dual role to both induce CDT and act as a stimulator of STING agonists, thereby enhancing the effectiveness of a vaccine adjuvant.^[^ ^19^ ^]^ To confirm the activation of the STING pathway by AMOPs, we used western blot analysis to assess the expression of phosphorylated TANK‐binding kinase 1 (pTBK1) and phosphorylated interferon regulatory factor 3 (pIRF3), the key transcription factors in the cGAS‐STING pathway. Figure 3F shows discernible bands for p‐STING, p‐TBK1, and p‐IRF3 in groups G3, G4, G6, and G7, signifying that Mn^2+^ is the principal activator of the STING pathway. Notably, the bands in the western blot for the G7 group were more pronounced than those in other groups, implying that DNA damage, augmented by the PTT‐enhanced CDT, further contributes to the STING pathway activation. The up‐regulation of IFN‐β secretion also showed the same trend (Figure S14, Supporting Information). Thus, AMOPs‐mediated therapy effectively induced ICD and activated the STING signaling pathway in tumor cells. These results demonstrate that AMOPs‐mediated therapy exhibited both antigenic and adjuvant properties, meeting the essential prerequisites for functioning as an in situ cancer vaccine.

### 2.4 Antitumor Effect of AMOPs‐Mediated Therapy in Vivo

The photoacoustic (PA)‐imaging capabilities of AMOPs were confirmed to gain insight into the in vivo enrichment behavior (Figure S15, Supporting Information). Notably, upon interaction with GSH, the PA signal of AMOPs was markedly intensified and maintained a positive correlation with the concentration of AMOPs. Driven by the superior GSH‐induced PA imaging of AMOPs observed in vitro, the validation imaging enrichment of the targeted tumor via intravenous injection was pursued. AMOPs were administered intravenously to mice with 4T1 tumors, and PA images were captured at intervals of 1, 4, 8, 12, and 24 h postinjection. Compared with that in the control group, a gradual enhancement in brightness at the tumor sites within the PA images was noted during the 1–8 h period postinjection. A progressive decrease in PA signal intensity of the tumor was observed between 8 and 24 h with further blood circulation and metabolic processes. The PA signal from the tumor imaging peaked at 8 h after the intravenous administration of AMOPs, suggesting an effective accumulation of AMOPs in the tumor area, likely due to the enhanced permeability and retention effect. This accumulation of NPs, predominantly occurring within the initial 8 h, provides precise temporal guidance for the PTT.

To further clarify the biodistribution and metabolic behavior of AMOPs in vivo, the organ enrichment and metabolism of Au elements were characterized using an inductively coupled plasma optical emission spectrometer (ICP‐OES). Based on the PA imaging, the distribution of AMOPs was first analyzed at 8 h after injection. It was found that 40.630 ± 7.247% of AMOPs were enriched in the liver, 8.223 ± 1.587% in the spleen, and 6.923 ± 0.815% in the tumor (Figure S16, Supporting Information). However, the enrichment of AMOPs in the heart, lungs, and kidneys was almost negligible. Subsequently, the metabolic behavior of AMOPs in vivo was analyzed (Figure S17, Supporting Information). The results indicate that AMOPs can be efficiently enriched to tumor regions and gradually metabolized out of the body.

**Figure 4:**
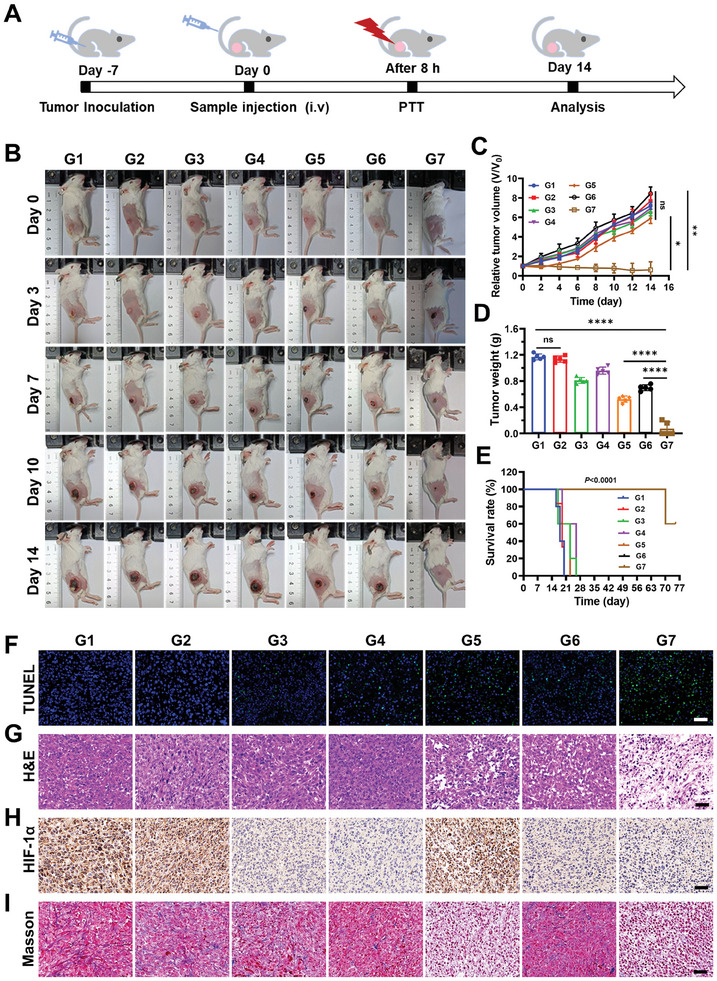
Anti‐tumor effect of AMOPs‐mediated therapy in vivo A) Schematic illustration of treatment schedules of 4T1 tumor‐bearing mouse model. B) Representative photograph of the mice after treatment at 0, 3, 7, 10 and 14 days. C) Relative tumor volume of the mice in each group during the 14 days after treatment (*n* = 5). D) Tumor weight of the mice in each group after treatment 14 days (*n* = 5). E) Survival curves of the mice in different treatment groups (*n* = 5). (F) TUNEL staining, G) H&E staining, H) immunohistochemistry staining of the expression of HIF‐1α, and I) Masson Trichrome staining in the tumor region after different treatments. Scale bar:50 µm. Groups are as follows (G1: PBS; G2: Au NPs; G3: MnO_2_ NPs; G4: AMOPs; G5: Au NPs + Laser; G6: MnO_2_ NPs + Laser; G7: AMOPs + Laser). Data are presented as the mean ± SD. Significance was calculated via an unpaired *t*‐test. (*ns*, no significant; ^*^ *p* < 0.05; ^**^ *p* < 0.01; ^***^ *p* < 0.001; ^****^ *p* < 0.0001).

Subsequently, the antitumor therapeutic efficacy of the laser‐triggered AMOPs in situ cancer vaccine was explored in a subcutaneous tumor model in mice. Mice carrying 4T1 tumors were randomly divided into seven groups: (G1) PBS, (G2) Au NPs (15 mg kg^−1^), (G3) MnO_2_ NPs (15 mg kg^−1^), (G4) AMOPs (15 mg kg^−1^), (G5) Au NPs + Laser, (G6) MnO_2_ NPs + Laser, and (G7) AMOPs + Laser. At 8 h postinjection, the tumor region of the mice was irradiated with a laser (808 nm, 1.0 W cm^−2^) for 8 min, followed by a 14‐day monitoring period (**Figure** **4**A). Compared with that in other groups, the tumor growth in group G7 was significantly hindered, with near elimination of the tumors (Figure 4B–D; Figures S18 and S19, Supporting Information). Notably, 80% of the mice in the AMOPs + Laser treatment group exhibited a tumor‐free survival rate (Figure 4E). Furthermore, no significant changes in body weight, temperature, or any apparent lesions in the major organs were observed in any group during the post‐treatment observation period (Figures S20–S22, Supporting Information), confirming the favorable biocompatibility and safety of the different treatment modalities. Tumor tissue staining with dUTP nick end labeling (TUNEL) (Figure 4F) revealed significant apoptosis of tumor cells in the G7 group compared with that of other treatments. Hematoxylin and eosin (H&E) staining (Figure 4G) of the tumor tissues also indicated extensive death of tumor cells in the AMOPs + Laser group, confirming the potent tumoricidal capability of the activated AMOPs as an in vivo vaccine.

Given that hypoxia is a primary characteristic of solid tumors, hypoxia‐inducible factor 1‐alpha (HIF‐1α) serves as a key regulatory factor produced by tumor hypoxia.^[^ ^20^ ^]^ Consequently, the expression levels of HIF‐1α in the tumor regions of different groups were assessed via immunohistochemistry. A significant reduction in HIF‐1α expression was observed in groups G3, G4, G6, and G7 (Figure 4H), suggesting that the MnO_2_ can catalyze the decomposition of H_2_O_2_ in the tumor region, triggering the release of O_2_ and thereby alleviating the hypoxic state within TME and boosting the function of CTLs. Moreover, the physical scaffold of the TME is composed of collagen, which causes the dense accumulation of the ECM, and is a significant barrier faced by tumor immunotherapy.^[^ ^21^ ^]^ Consequently, the degradation of collagen helps in normalizing the immune‐suppressed TME, further enabling tumor infiltration by CTLs. Previous studies have shown that PTT can degrade tumor collagen.^[^ ^22^ ^]^ To confirm this phenomenon, Masson's trichrome staining of tumor tissues was performed. As depicted in Figure 4I, a notable loosening of collagen was observed in the tumor tissues of groups G5 and G7, indicating that PTT induced the degradation of collagen within the tumors and would thereby favor CTLs in crossing the tumor stromal barrier and infiltrating the tumor. Therefore, the laser‐triggered AMOPs can alleviate the hypoxic state and degrade the ECM to enhance the quantity and quality of CTLs in the tumor areas, thereby ensuring that the vaccine exerts a robust antitumor effect.

### 2.5 Immune Activation Ability of AMOPs‐Mediated Therapy

**Figure 5:**
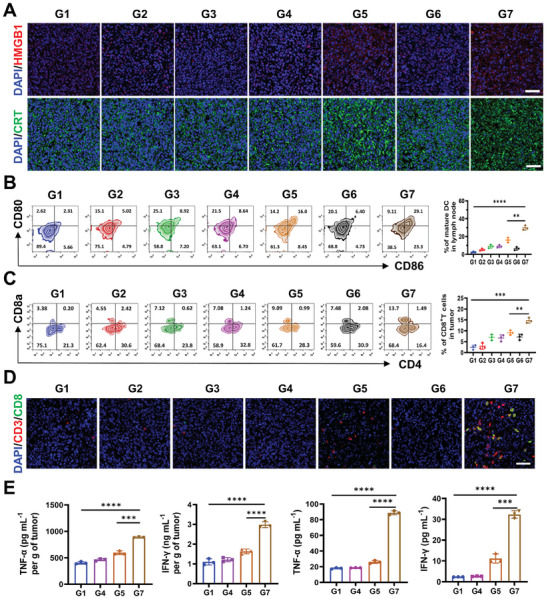
Immune activation ability of AMOPs‐mediated therapy. A) Immunofluorescence of HMGB1 and CRT in the tumor tissues after different treatments. Scale bar: 50 µm. B) Representative flow cytometric analysis showing the percentages of matured DCs (CD11c^+^ CD80^+^, and CD86^+^) in the lymph nodes and the quantification of matured DCs. (*n* = 3). C) Representative flow cytometric analysis showing the percentages of CTLs (CD3^+^ CD4^+^, and CD8a^+^) in the tumor tissues and the quantification of CTLs. (*n* = 3). D) Immunofluorescence of CD8^+^ T cells in the tumor tissues after different treatments. Scale bar: 50 µm. E) The ELISA test of IFN‐γ and TNF‐α in tumor tissue and serum. (*n* = 3). (Groups: G1: PBS; G2: Au NPs; G3: MnO_2_ NPs; G4: AMOPs; G5: Au NPs + Laser; G6: MnO_2_ NPs + Laser; G7: AMOPs + Laser). Data are presented as the mean ± SD. Significance was calculated via unpaired *t*‐test (^**^ *p* < 0.01; ^***^ *p* < 0.001; ^****^ *p* < 0.0001).

The AMOPs vaccine has achieved encouraging success in subcutaneous tumors, and we have further explored its immunostimulatory effects. First, immunofluorescence staining was employed to analyze the induction of ICD across treatment groups. The release of HMGB1 and the exposure of CRT observed in the tumors of groups G5 and G7 were consistent with the results from the in vitro analysis, confirming the effective induction of ICD in vivo (**Figure** **5**A). Subsequently, AMOPs‐mediated activation of the immune system was validated by assessing the maturation of DCs within the mice in different groups. The G7 group exhibited the highest maturation rate of DCs, reaching 29.1%, which was significantly higher than the rate of 2.31% observed in the control group (Figure 5B). This indicates that the AMOPs vaccine can effectively promote DC differentiation, thereby stimulating an adaptive immune response. Further analysis was conducted using flow cytometry to detect the proportion of CD8^+^ T cells (Figure 5C). The G7 group exhibited a CD8^+^ T‐cell ratio of 13.7%, a 4.05‐fold increase compared with that of the G1 group. In parallel, immunofluorescence staining of the tumor tissue revealed that the G7 tumor site had the most abundant infiltration of CD8^+^ T cells (Figure 5D), which corroborated the flow cytometry results. This result was attributed to both the immune activation caused by ICD and the enhancement of infiltration and function of CTLs via improved O_2_ supply and ECM clearance. Then, cytokine levels in both tumor tissue and serum were measured using enzyme‐linked immunoassay (ELISA) (Figure 5E). Compared with that in other groups, G7 treatment significantly increased the levels of interferon‐gamma (IFN‐γ) and tumor necrosis factor‐alpha (TNF‐α). Last, to confirm that the immune activation resulted from the efficacy of the AMOPs vaccine rather than other indirect biological processes, the OVA‐4T1 mouse model was utilized to characterize the proportion of antigen‐specific T cells in the spleen of mice from Group G1 and G7 at 7 days post‐vaccination. As shown in Figure S23 (Supporting Information), the percentage of T cells with CD3^+^CD8^+^OVA Tetramer^+^ in the spleen was significantly higher in the G7 group than in the G1 group. This suggests that our vaccination indeed produced antigen‐specific T cells, further demonstrating the effectiveness of the AMOPs vaccine. These data confirm that AMOPs, as an in situ cancer vaccine, not only effectively kill tumor cells but also trigger a robust antitumor immune response, laying a solid foundation for subsequent success in metastatic and rechallenge models.

### 2.6 Long‐Term Antitumor Memory Immune Response Triggered by AMOPs‐Mediated Therapy in Recurrence and Metastasis Model

Tumor recurrence and metastasis are pivotal factors contributing to mortality in cancer patients.^[^ ^23^ ^]^ As AMOPs had demonstrated an outstanding antitumor immune efficacy in subcutaneous tumor models, we then thoroughly evaluated their potential for long‐term immune memory and its capacity to combat recurrence and metastasis.

**Figure 6:**
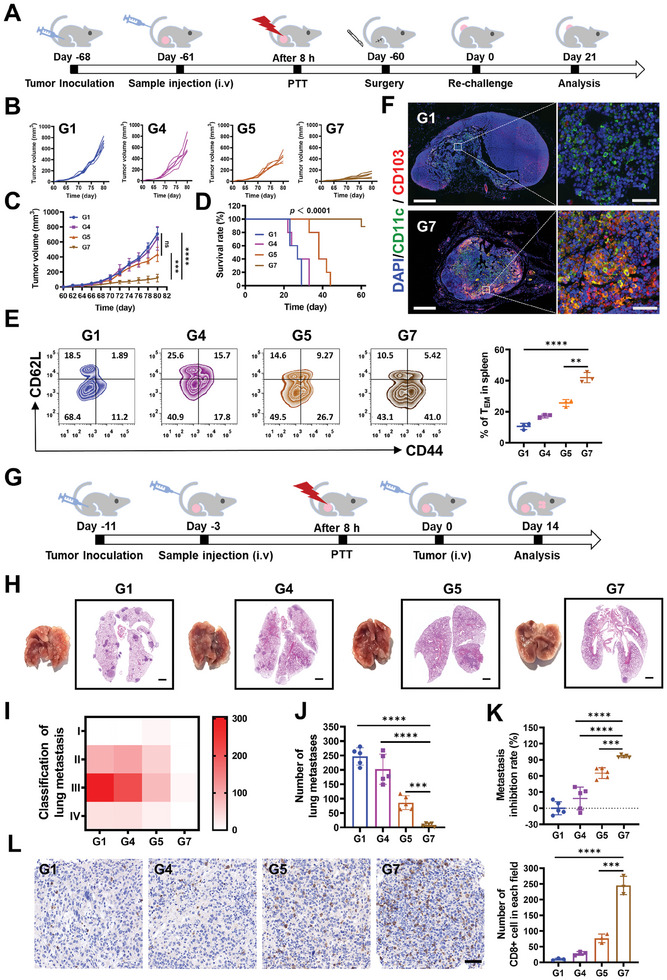
Long‐term antitumor memory immune response triggered by AMOPs‐mediated therapy in recurrence and metastasis model. A) Schematic of tumor rechallenge assay schedules of 4T1 tumor‐bearing mouse model. Different treatments are (G1) PBS, (G4) AMOPs (15 mg kg^−1^), (G5) Au NPs + Laser irradiation (15 mg kg^−1^; 808 nm 1.0 W cm^−2^, 8 min), and (G7) AMOPs + Laser irradiation (15 mg kg^−1^; 808 nm 1.0 W cm^−2^, 8 min). B) Tumor growth kinetics of tumor rechallenge assay (*n* = 5). C) Relative rechallenge tumor volume of the mice in each group after rechallenge 20 days (*n* = 5). D) Survival curves of different treatment groups within 60 days (*n* = 5). E) Representative flow cytometric analysis of memory T cells within the spleen after tumor rechallenge assay. (*n* = ). F) Representative images of immunofluorescent staining of CD11c^+^ and CD103^+^ DC cells in mouse lymph nodes with different treatments (blue, cell nuclei staining; green, CD11c^+^ staining, red, CD103^+^ staining); scale bar: 1 mm (left panels), 50 µm (right panels). G) Schematic of lung metastasis assay schedules of 4T1 tumor‐bearing mouse model. H) Representative images and H&E staining of lungs harvested from the metastatic 4T1 tumor‐bearing mice on day 14; scale bar: 500 µm. I) Heat map of tumor nodules in lungs collected on day 14. (*n* = 5). J) Numbers of tumor nodules in the lungs collected on day 14 (*n* = 5). K) Metastasis inhibition rate of different groups. L) Representative immunohistochemical images of CD8^+^ cells (brown) in lung tissues collected on day 14 and the quantitation of CD8^+^ cells in each field (*n* = 3); scale bar: 50 µm. Data are presented as the mean ± SD. Significance was calculated via unpaired *t*‐test (ns, not significant; ^*^ *p* < 0.05; ^**^ *p* < 0.01; ^***^ *p* < 0.001; ^****^ *p* < 0.0001).

First, 4T1 tumor‐bearing mice were treated in the groups previously delineated: G1 (PBS as the control group), G4 (AMOPs as the CDT group), G5 (Au NPs + Laser as the PTT group), and G7 (AMOPs + Laser as the vaccine group). At 7 days posttreatment, the subcutaneous tumors were surgically excised. At the 60‐day mark, the mice were rechallenged with 4T1 cells on the contralateral flank to evaluate the immune memory induced by the AMOPs vaccine and its role in preventing tumor growth recurrence (**Figure** **6**A). As shown in Figure 6B,C and Figure S24 (Supporting Information), the comparison between group G4 and the control group G1 did not demonstrate a significant tumor suppression effect. Group G5 showed a moderated retardation of the rechallenged tumor growth, which may be attributed to the ICD induced by PTT. Encouragingly, mice in group G7 exhibited a robust inhibition during tumor rechallenge, significantly delaying tumor growth and presenting a satisfactory vaccine effect. Concurrently, at the end of the study, mice in group G7 showed a favorable survival rate of 80% (Figure 6D). The prophylactic efficacy against tumor recurrence manifested by the AMOPs vaccine is likely attributable to an elicited immune memory response. To elucidate this immunological mechanism, we quantified the splenic memory T‐cell subsets via flow cytometric analysis. The proportion of effector memory T cells in group G7 was significantly higher than that in other groups, reaching 41% (Figure 6E). To gain a more profound understanding of the vaccine effect elicited by AMOPs, we isolated lymph nodes from mice in groups G1 and G7 at the end of the treatment and characterized the cDC1 cells (CD11c^+^ and CD103^+^) within the lymph nodes through immunofluorescence staining. The number of cDC1 cells was significantly higher in the lymph nodes of group G7 than in group G1 (Figure 6F), suggesting a substantially higher efficiency in tumor antigen presentation in the G7. This finding further corroborates the efficacy of AMOPs as in situ cancer vaccines.

Next, the suppressive effect of the AMOPs vaccine on tumor metastasis was validated using groups G1, G4, G5, and G7. On the third day posttreatment, 4T1 cells were injected via the tail vein to simulate circulating tumor cells. After another 14 days, mouse pulmonary tissues were collected and assessed for metastatic development (Figure 6G). H&E staining of the lung tissues revealed extensive metastases in groups G1 and G4, with an ameliorated condition in group G5, consistent with previous trends shown in other models (Figure 6H). Notably, group G7 exhibited significant inhibition of tumor metastasis, with a substantially reduced number of metastases than that present in the other groups, indicating the efficacy of the AMOPs vaccine in countering tumor metastasis (Figure 6I–K). Moreover, an analysis of immune infiltration in metastatic tumors across the groups revealed that the degree of CTL infiltration in the metastatic foci of group G7 was markedly higher than that in other groups (Figure 6L), further demonstrating the potent immune surveillance function triggered by the AMOPs vaccine. Thus, AMOPs vaccination not only inhibited the subcutaneous tumor and improved the immune microenvironment but also indirectly influenced the immune infiltration of metastatic foci through an immune memory effect.

## 3 Conclusion

Here, we proposed a vaccination strategy for synergistic ICD induction with optimized TME to achieve enhancement of CTLs function and infiltration and induce potent antitumor immune effects. The AMOPs vaccine effectively catalyzed the decomposition of H_2_O_2_ into O_2_ within the TME, alleviating hypoxia and restoring the function of CTLs. Additionally, high concentrations of GSH in tumor cells activated the PTT properties of AMOPs, leading to ICD and degradation of the ECM, which facilitated CTLs infiltration. Importantly, AMOPs also activated the STING pathway through the released Mn^2+^, enhancing the adjuvanticity of the vaccine. The synergistic effects of these mechanisms enabled the AMOPs vaccine to robustly inhibit subcutaneous tumor growth, establish strong immunological memory, and effectively prevent tumor recurrence and metastasis. Additionally, we were delighted to find that the AMOPs vaccine not only enhanced the infiltration and function of CTLs but also significantly increased the accumulation of cDC1 in lymph nodes. This observation highlighted the crucial role of our strategy in promoting dendritic cell migration and improving antigen presentation. Thus, our study provides a new insight into the development of in situ cancer vaccines and cancer immunotherapy.

## 4 Experimental Section

### Materials

All chemical reagents were used without further purification. Chloroauric acid (HAucl_4_·4H_2_O) was purchased from Innochem (Beijing). Sodium citrate was purchased from Tianjin Tianli Chemical Reagents Co., Ltd. Potassium permanganate was purchased from Sigma‐Aldrich. The culture medium and fetal bovine serum were purchased from Gibco Life Technologies. Flow cytometry antibodies were purchased from BioLegend Ltd. Western‐blot antibodies were purchased from Cell Signaling Technology, Inc. All kinds of kits were purchased from Beyotime Biotechnology. 4T1 cells, DC 2.4 cells, and L‐02 cells were purchased from Zhongqiaoxinzhou (Shanghai, China). The BALB/c female mice were provided by the Animal Center of the Fourth Military Medical University (Xi'an, China). The animal protocols of this study were reviewed and approved by the Institutional Animal Care and Use Committee of the Fourth Military Medical University (approval number: 20220312).

### Synthesis of AMOPs

AMOPs were synthesized in two steps. First, HAuCl_4_ (5 mg, 1 wt.%) was dissolved in ultrapure water (50 mL) and brought to a boil under stirring. Sodium citrate solution (1 mL, 1 wt.%) was then rapidly added. The solution was allowed to continue boiling with reflux condensation for 15 min before being cooled to room temperature in an ice bath, thus successfully completing the preparation of Au nanoparticles (Au NPs). Subsequently, a solution of KMnO_4_ (15 mL, 10 mm) was added dropwise to the solution of Au nanoparticles (48 mL) under constant stirring for 1 h. This mixture was then transferred to a constant temperature water bath at 80 °C for 30 min, followed by cooling to room temperature in an ice bath. The synthesized AMOPs were finally collected by centrifugation and redispersed in ultrapure water for further use.

### Oxygen Generation

MnO_2_ and AMOPs at the same concentration (100 µm in terms of Mn) were dissolved in H_2_O_2_ solution (100 µm), and then the oxygen content was detected using an oxygen dissolving meter.

### Characterization of Photothermal Performance

The 10 mm of GSH, respectively containing different concentrations (0, 25, 50, 75, and 100 µg mL^−1^) of AMOPs, were irradiated by 808 nm laser at 1.0 W cm^−2^ power density for 8 min, and the temperature change was recorded using an infrared thermal imager. Then, 100 µg mL^−1^ AMOPs respectively dispersed into water and 10 mm of GSH, the mixture was irradiated, and the temperature change was recorded as described above. Finally, 10 mm of GSH containing 100 µg mL^−1^ AMOPs were irradiated as described above for 500 s and then stopped irradiation for the same time, repeating above four times. Meanwhile, the temperature change was recorded.

### Hydroxyl Radical (•OH) Generation In Vitro

A buffer solution containing 25 mm NaHCO_3_ and 5% CO_2_, supplemented with AMOPs ([Mn] = 0.3 mm) and GSH (1.0 mm), was subjected to shaking for 15 min. Following centrifugation, methylene blue (MB) at a concentration of 5 µg mL^−1^ and H_2_O_2_ at 5 mm were introduced to the supernatant. Subsequently, the mixture was incubated at 25 °C for 30 min, after which the ·OH‐induced absorbance alteration of MB at 665 nm was recorded. Furthermore, to examine the effect of temperature on the reaction, the procedure was replicated with incubation temperatures set at 37 and 46 °C. The ·OH‐induced absorbance changes of MB at 665 nm were measured at various time points, specifically after 0, 15, 30, 45, 60, 75, and 90 min of incubation.

### Characterization of Photoacoustic (PA) Imaging

To assess the PA imaging performance of AMOPs solutions both in the absence and presence of GSH, solutions with varying concentrations of AMOPs (30, 60, 90, 120, and 150 µg mL^−1^) were prepared. The imaging was carried out using a multispectral photoacoustic tomography system (MSOT: inVision 128; iThera Medical), with PBS serving as the control.

### Cell Experiments

4T1 cells and L‐02 cells were cultured in RPMI‐1640 medium with 10% fetal bovine serum (FBS) and 1% penicillin‐streptomycin. All cells were cultured at 37 °C in a humidified 5% CO_2_ Incubator. 4T1 cells were chosen as models in all the following cell experiments to evaluate the function and properties of AMOPs. Parameters of laser irradiation are 1.0 W cm^−2^, λ = 808 nm. The duration of laser exposure is precisely 8 min.

### Cytotoxicity Assay

4T1 cells and L‐02 cells were inoculated into 96‐well plates at a density of 5 × 10^3^ cells per well and incubated for 24 h. Subsequently, the cells were treated with various concentrations (0, 12.5, 25, 50, 100, and 200 µg mL^−1^) of AMOPs. After an incubation period of 24 h, cell viability was assessed utilizing the MTT assay.

### Induction of Immunogenic Cell Death (ICD) In Vitro

4T1 cells were seeded into 24‐well plates at a density of 3 × 10^3^ cells per well and incubated for 24 h. Treatment with Au NPs, MnO_2_ NPs, or AMOPs was administered to the cells, which were then exposed to an 808 nm laser at a power density of 1.0 W cm^−2^ for 8 min, or left unexposed, with PBS serving as the control. Following this, a further incubation period of 12 h was allowed. The cells were washed three times with PBS. For the purpose of immunofluorescence detection of CRT or HMGB1 expression, the cells underwent fixation with 4% paraformaldehyde, followed by incubation with either anti‐CRT antibody conjugated with FITC or anti‐HMGB1 antibody conjugated with PE for 30 min. Subsequent staining with DAPI for 10 min was performed, and the cells were then observed under an inverted fluorescence microscope. In the ATP release assay, the culture mediums were not subjected to washing with PBS prior to harvesting. The supernatants were collected via centrifugation at 400 g for 5 min. Measurement of the extracellularly released ATP content was conducted using an ATP Assay Kit, in accordance with the manufacturer's instructions.

### In Vivo Evaluation of Photothermal Performance

4T1 cancer‐bearing BABL/c female mice were randomly divided into four groups. After the tumor volume reached ≈100 mm^3^, the mice were intravenously administered by Au NPs, MnO_2_ NPs, and AMOPs solution, PBS as control. All dose was 15 mg kg^−1^. The mice were then irradiated with laser for 8 min (808 nm, 1.0 W cm^−2^) at 8 h postinjection. Meanwhile, the thermal images and temperatures were monitored by an infrared thermal imager.

### Characterization of the Antitumor Properties In Vivo

In order to establish the mouse tumor model, 4‐week‐old BALB/c female mice were subcutaneously injected with 5 × 10^5^ 4T1 cells on the right hind limb. Upon the tumor volumes reaching 100 mm^3^, the mice were randomly assigned into seven groups (*n* = 5) and received treatments as follows: (G1) PBS, (G2) Au NPs (15 mg kg^−1^), (G3) MnO_2_ NPs (15 mg kg^−1^), (G4) AMOPs (15 mg kg^−1^), (G5) Au NPs + Laser (Au: 15 mg kg^−1^, laser: 808 nm, 1.0 W cm^−2^, 8 min), (G6) MnO_2_ NPs + Laser (MnO_2_: 15 mg kg^−1^, laser: 808 nm, 1.0 W cm^−2^, 8 min), and (G7) AMOPs + Laser (AMOPs: 15 mg kg^−1^, laser: 808 nm, 1.0 W cm^−2^, 8 min), respectively. The tumor volumes, body weights, and body temperatures of the mice were recorded. The tumor volumes were calculated using the formula: tumor volume = 0.5×width^2^×length. The tumor inhibition rate was defined as the ratio of the tumor weight in the experimental group to that in the blank control group at the end of the experiment.

### Antitumor Immune Response Analysis

The analysis of immune cell infiltration was studied by immunofluorescence assay and flow cytometry. For flow cytometry immunofluorescence assay, prepare tumor tissue into slices and stain with anti‐CD3 and anti‐CD8 antibodies. For flow cytometry, the tumors and lymph nodes were collected. To analyze the DC cells (CD11c^+^, CD80^+^, CD86^+^), the lymph nodes in mice were obtained to extract lymphocytes, and stained with anti‐CD11c‐FITC, anti‐CD80‐APC, and anti‐CD86‐Cy5.5 antibody. For analyzing the tumor‐infiltrating lymphocytes, the isolated tumors were cut into small pieces, and the small pieces were submerged in collagenase IV (0.5 mg mL^−1^) with DNase I (0.1 mg mL^−1^) for 3 h at 37 °C. The acquired suspensions were filtered and the single cells were stained with fluorescence‐labeled antibodies (CD3^+^ CD4^−^ CD8^+^ T cells, CD4^+^ CD44+ CD62L^−^ memory T cells) for 30 min at 4 °C, measured by flow cytometer. Anti‐CD3‐FITC, anti‐CD4‐PE‐Cy7, anti‐CD8‐APC, anti‐CD62L‐PE, and anti‐CD44‐FITC were diluted by the manufacturer's direction. Moreover, 7 days after treatment, the blood samples of mice were centrifuged to obtain the serum, and the cytokine in serum, containing IFN‐γ, IFN‐β, and TNF‐α were measured using ELISA kits by the manufacturer's methods.

### Antitumor Immune Response Memory Analysis

To demonstrate the establishment of immune memory, an in vivo therapeutic experiment was re‐conducted. When the tumor volume reached 100 mm^3^, the tumor‐bearing mice were randomized into four groups (*n* = 5) and treated with (G1) PBS, (G4) AMOPs (15 mg kg^−1^), (G5) Au NPs +Laser (Au: 15 mg kg^−1^, laser: 1.0 W cm^−2^, 8 min), (G7) AMOPs + Laser (AMOPs: 15 mg kg^−1^, laser: 1.0 W cm^−2^ 8 min), respectively. The tumors were removed after 2‐day treatment. Then, the tumor‐free mice were re‐challenged with 5 × 10^5^ of 4T1 cells at day 60 after the first tumor was removed. Measurements of tumor volumes and body weights of mice were performed every other day. Three‐week post tumor re‐challenge, mice were sacrificed, the spleens and lymph nodes were acquired and digested into the single cells, the single cells were stained with fluorescence‐labeled antibodies (CD4, CD44, CD62L) and then the memory lymphocytes were analyzed by FACS analysis. The lymph nodes were acquired for immunofluorescent staining(CD11c, CD103).

### In Vivo Tumor Metastasis Experiments

To establish the lung metastatic tumor model, 5 × 10^5^ 4T1 cells were subcutaneously injected into the right root of the hind limb of BALB/c mice (primary tumor). When the tumor volume reached 100 mm^3^, the mice were randomly divided into four groups (*n* = 5), and treated with (G1) PBS, (G4) AMOPs (15 mg kg^−1^), (G5) Au NPs +Laser (Au: 15 mg kg^−1^, laser: 1.0 W cm^−2^ 8 min), (G7) AMOPs + Laser (AMOPs: 15 mg kg^−1^, laser: 808 nm, 1.0 W cm^−2^, 8 min), respectively. After 2 days, 5 × 10^5^ 4T1 cells were injected into the tail intravenous of mice. After 2 weeks, mice were sacrificed, and the lung were collected and fixed with 4% paraformaldehyde, the amount of tumor metastatic nodules in the lung was counted, and then stained by H&E and assayed by immunohistochemical localization (CD8).

### Statistical Analysis

Statistical analyses were performed using GraphPad Prism software. Data are presented as mean ± standard deviation (SD), with error bars indicating SD. The mean and SD values were calculated from three or more independent experiments. Comparison between the two groups was made by Student's two‐tailed *t*‐test. Statistical significance was established as indicated ^*^ *p* < 0.05; ^**^ *p* < 0.01; ^***^ *p* < 0.001 and ^****^ *p* < 0.0001.

## Conflict of Interest

The authors declare no conflict of interest.

## Supporting information

Supporting Information

## Data Availability

The data that support the findings of this study are available from the corresponding author upon reasonable request.
