# Supplementary material for: Light‐Activated In Situ Vaccine with Enhanced Cytotoxic T Lymphocyte Infiltration and Function for Potent Cancer Immunotherapy
Source: Adv Sci (Weinh). 2024 Jul 2;11(33):2403158. doi: 10.1002/advs.202403158 (PMC11434106; doi:10.1002/advs.202403158)
Supplement: Supplementary file 1 — Supporting Information [file ADVS-11-2403158-s001.docx]

Supporting Information

Light-Activated In Situ Vaccine with Enhanced Cytotoxic T Lymphocyte Infiltration and Function for Potent Cancer Immunotherapy

Xian An, Zhuang Chen*, Yi Luo, Peng Yang, Zuo Yang, Tiannan Ji, Yajing Chi, Shuyuan Wang, Ruili Zhang*, Zhongliang Wang*, and Jianxiong Li*

**Supplementary Figures**


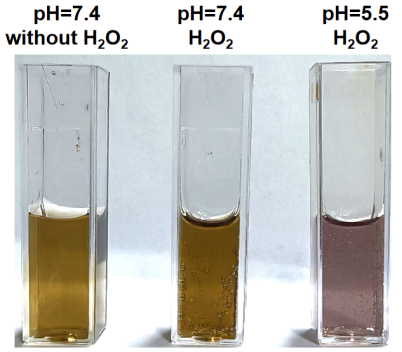


Figure S1. Photographs of AMOPs in H_2_O_2_ solutions with different pH values.


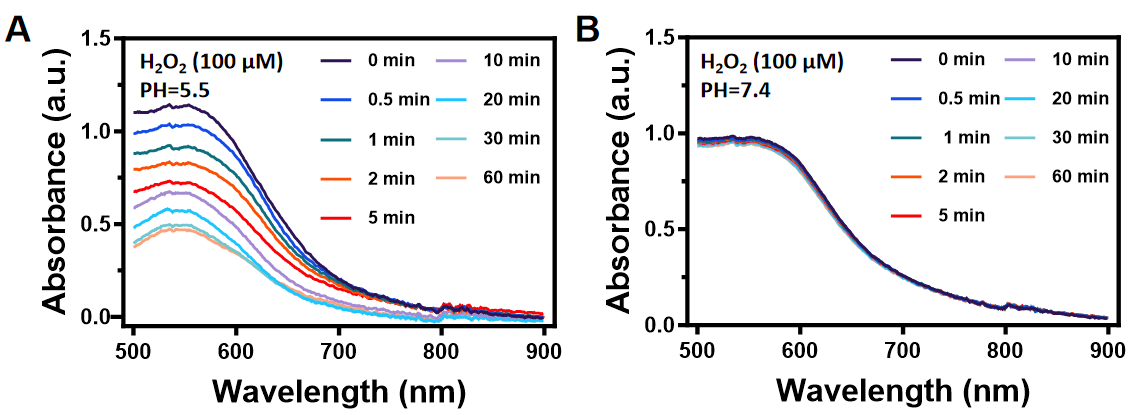


Figure S2. The absorption spectra of AMOPs in H_2_O_2_ solutions with different pH values.


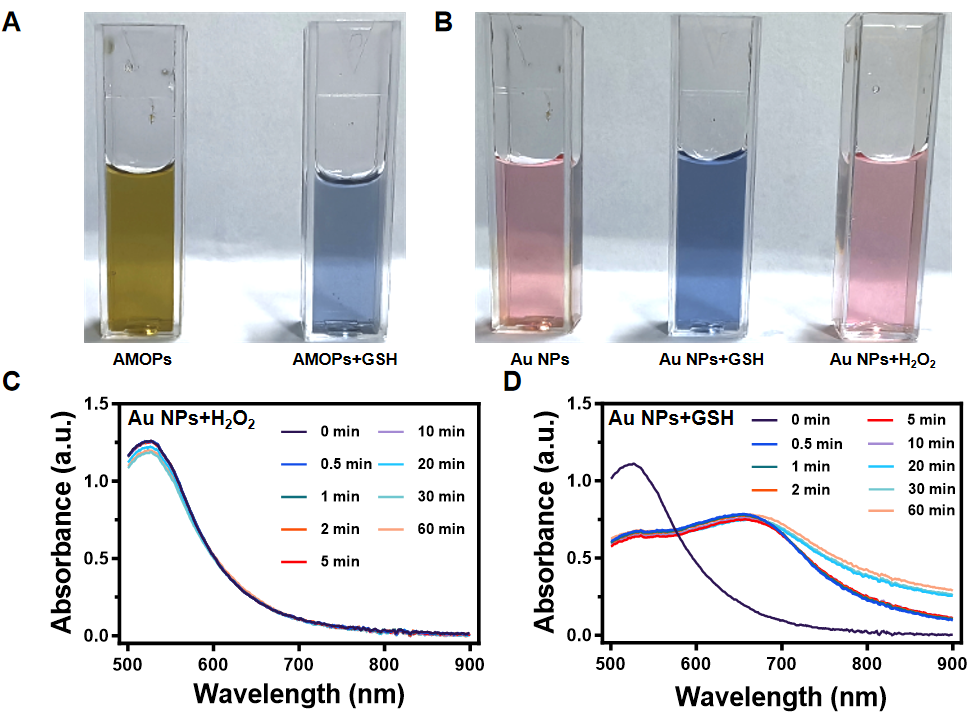


Figure S3. Behavior and absorption spectra of Au NPs in GSH and H_2_O_2_ solutions. (A) photograph of the AMOPs in GSH solution, (B) photograph of the Au NPs in GSH and H_2_O_2_ solutions. The absorption spectra of Au NPs (C) in H_2_O_2_ solutions and (D) in GSH solutions for different time incubation.


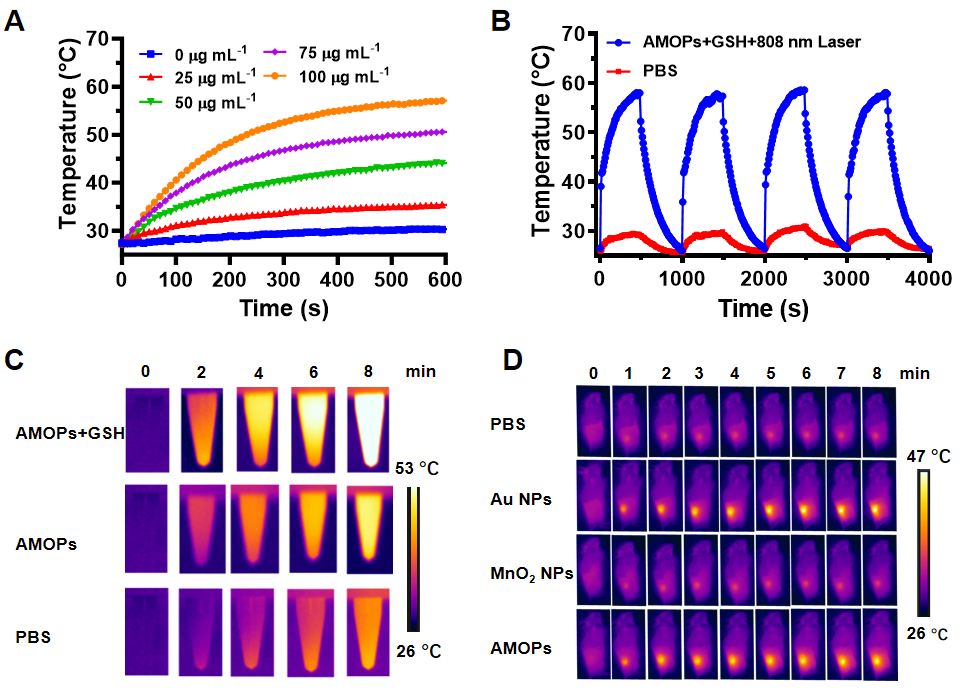


**Figure S4.** (**A**) Temperature variation of AMOPs with different concentrations after 808 nm NIR laser irradiate (1.0 W cm^−2^) for 10 min. (**B**) Temperature elevation curves of AMOPs over four cycles of 808 nm NIR laser on/off irradiation. (**C**) Thermal images of AMOPs in absence or presence of GSH (10 mM), PBS as control. (**D**) Thermal images of the mice in the PBS + Laser, Au NPs +Laser, MnO_2_ NPs + Laser and AMOPs + Laser groups before and after an 8 h intravenous injection of PBS, Au NPs, MnO_2_ NPs and AMOPs, respectively under 8 min of 808 nm laser irradiation (1.0 W cm^-2^).


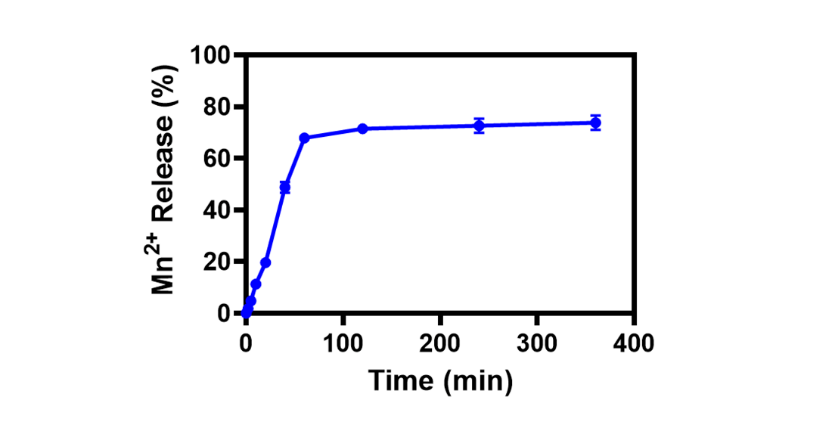


**Figure S5.** The Mn^2+^ release of AMOPs in GSH (10 mM) solution (*n* = 3).


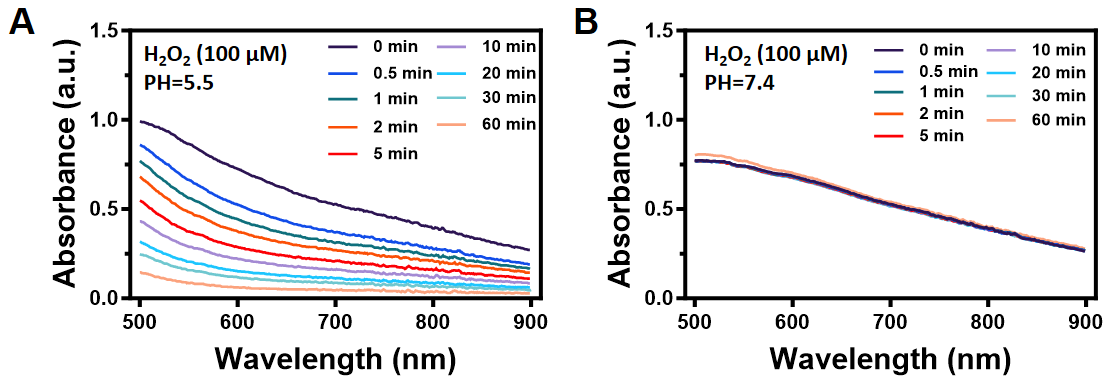


Figure S6. Degradation behavior of MnO_2_ NPs in H_2_O_2_ solutions of different pH. Changes in absorption spectra of MnO_2_ NPs in a H_2_O_2_ solution (A) at pH=5.5, and (B) at pH=7.4.


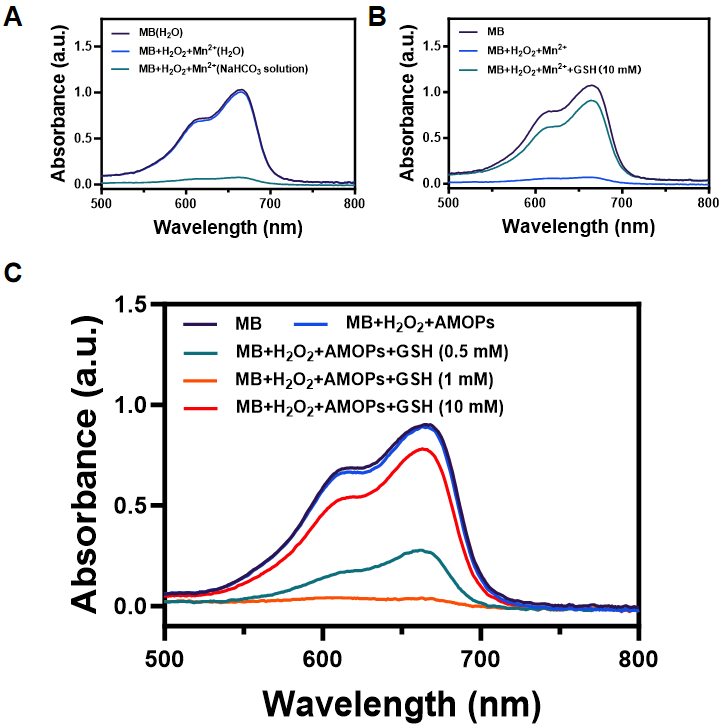


Figure S7. Characterization of the CDT process mediated by the AMOPs. (A) Necessary conditions for the Mn^2+^-mediated CDT process. (B) Ininhibitory effect of GSH on Mn^2+^-mediated CDT process. (C) Characterization of the CDT performance of AMOPs at different GSH concentrations.


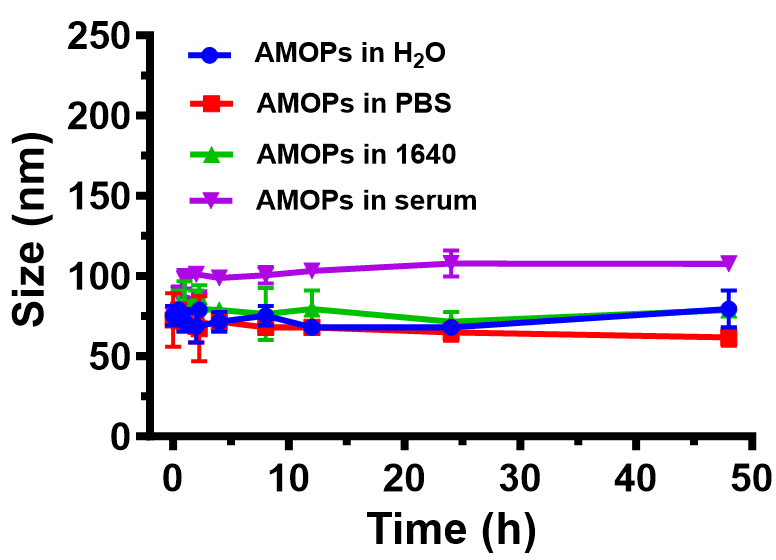


Figure S8. Changes in hydrodynamic diameter of AMOPs in water, PBS, culture medium and serum within 48 h (*n* = 3).

**
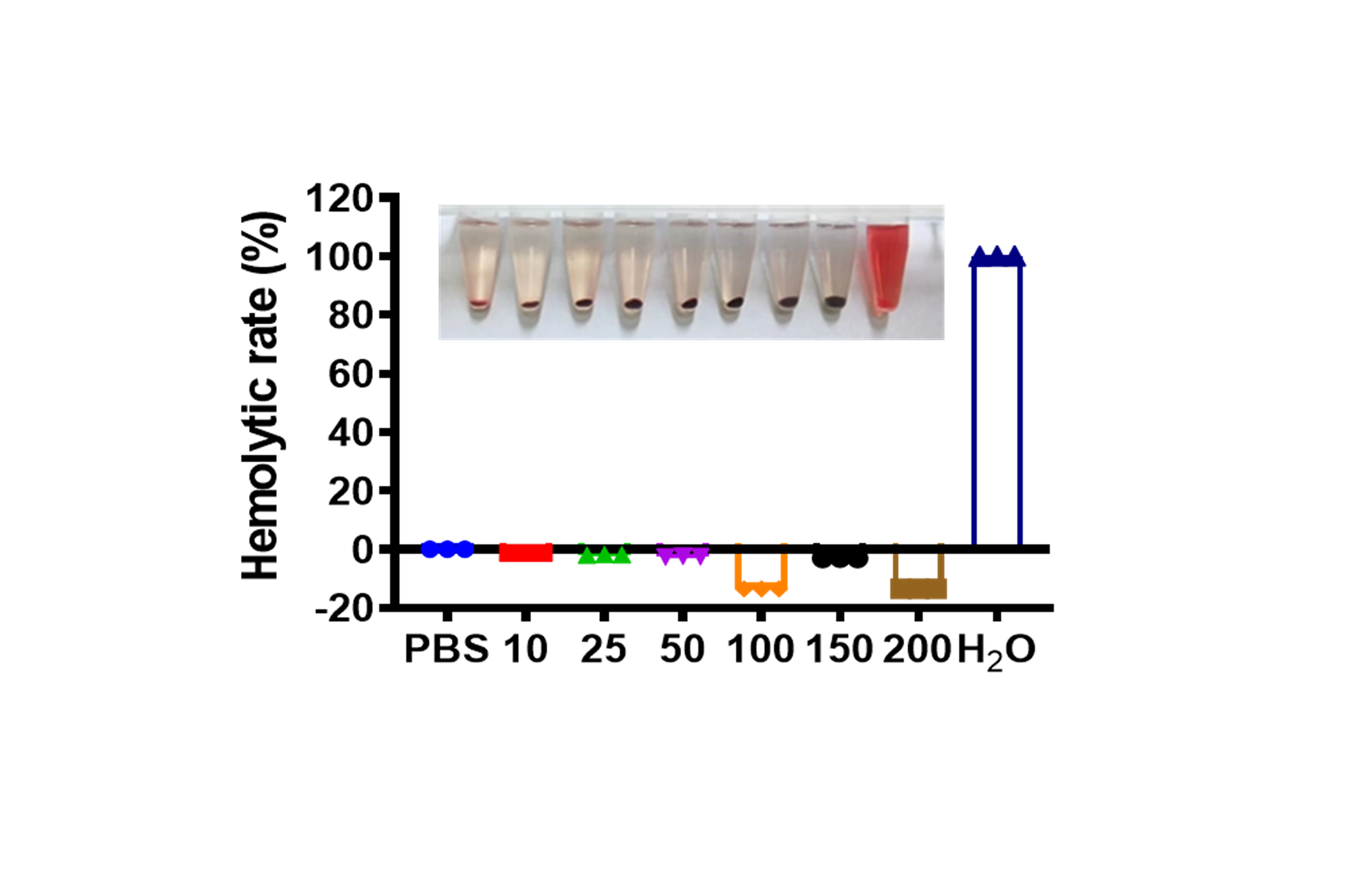
**

**Figure S9.** Hemolysis of different concentrations of AMOPs (*n* = 3).

.


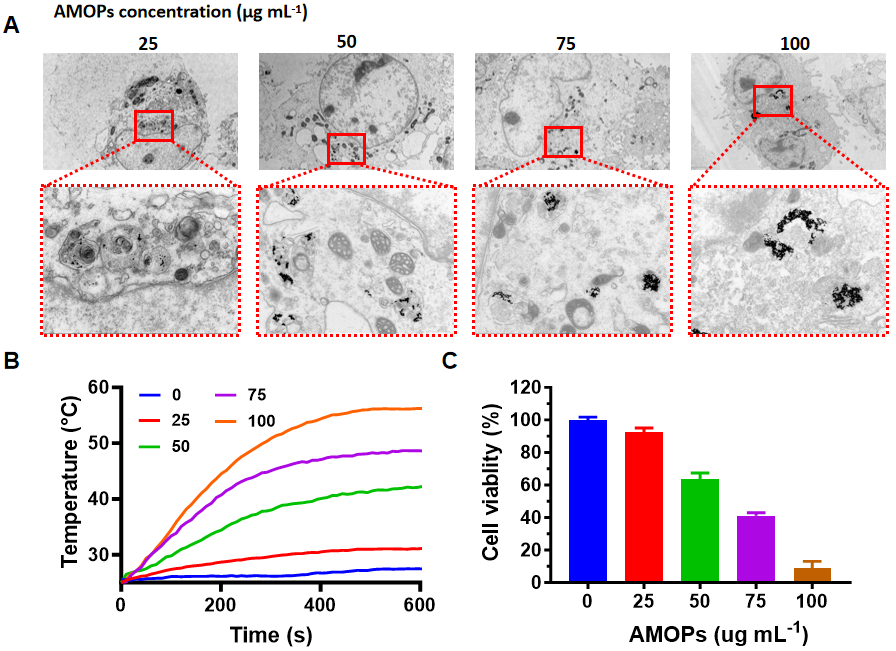


Figure S10. Characterization of the aggregation behavior and photothermal toxicity of AMOPs in 4T1 cells. (A) TEM images of 4T1 cells after incubation with different concentrations of AMOPs. (B) Photothermal heating curves of 4T1 cells after incubation with different concentrations of AMOPs (after removing the medium). (C) Cell Viability of 4T1 cells after PTT with different concentrations of AMOPs.


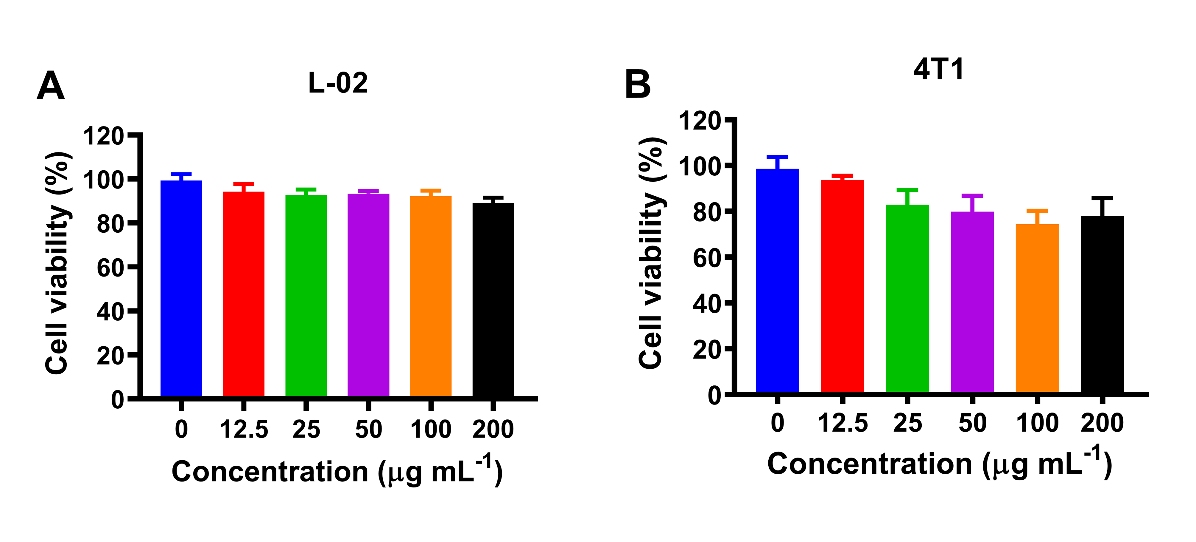


**Figure S11.** The viability of normal cells (L-02 cells) **(A)** or tumor cells (4T1 cells) **(B)** after treatment with different concentrations of AMOPs were measured using the MTT assay (*n* = 5).


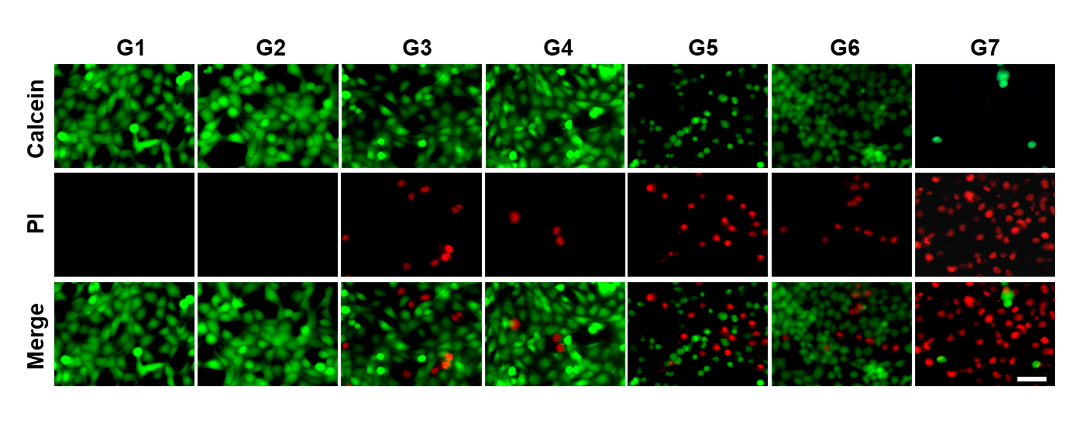


**Figure S12.** Live/dead cell staining at 12 h after treatment and laser irradiation for 4T1 cells. Scale bar: 50 µm.


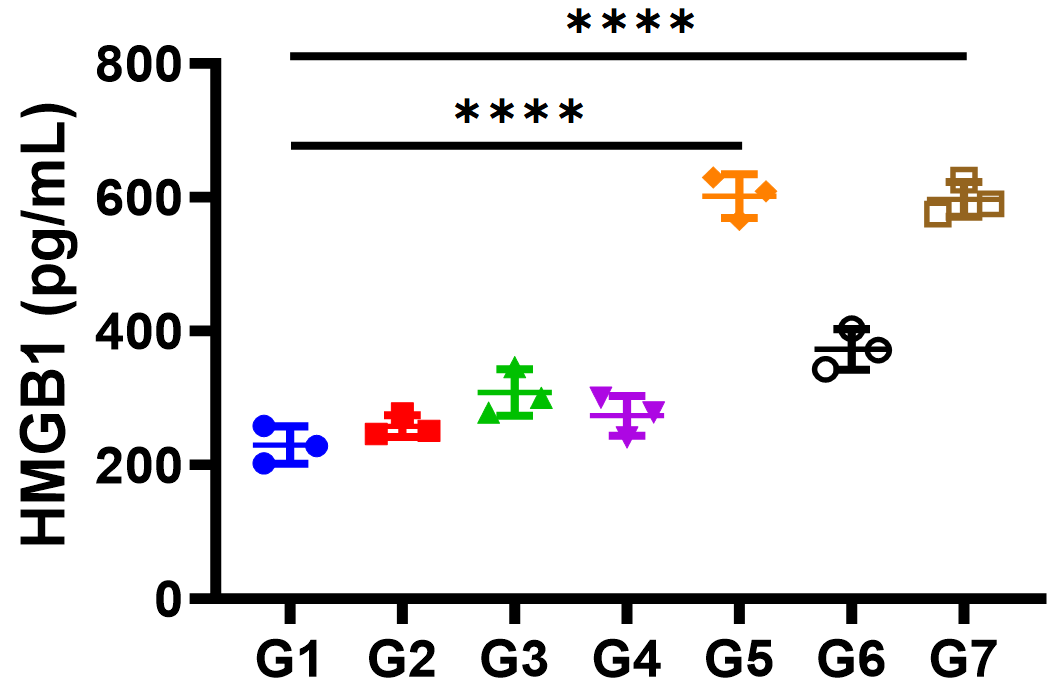


**Figure S13.** Release of HMGB1 after the different treatments (*n* = 3).

.


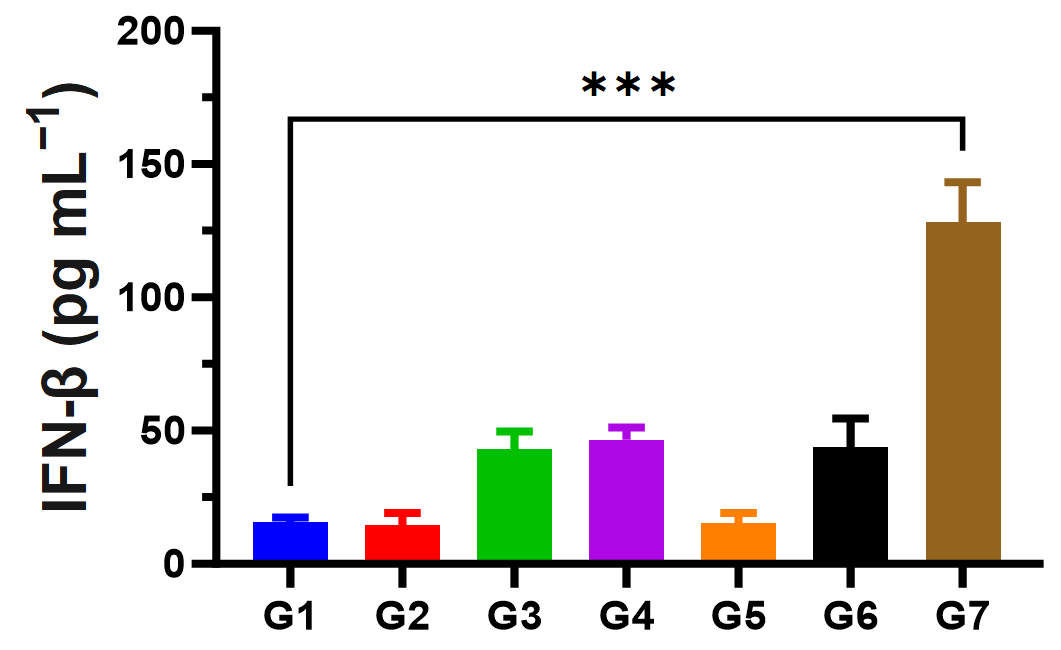


**Figure S14**. The IFN-β secretion from cocultured DC2.4 and 4T1 cells after different treatments (*n* = 3).

.


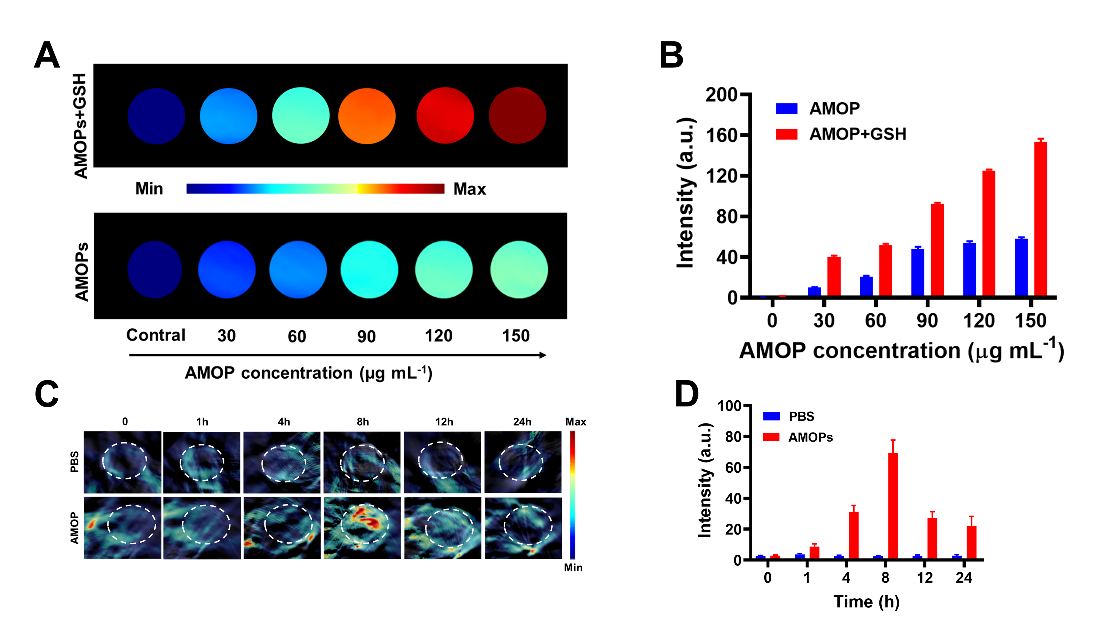


**Figure S15.** (**A**) Photoacoustic images of the PBS (control) and AMOPs with different Mn concentrations in the presence of GSH and (**B**) the corresponding signal intensity (*n* = 3). (**C**) Photoacoustic imaging of a tumor marked with white line ellipses before (control) and 1, 4, 8, 12, and 24 h after the intravenous injection of AMOPs, and (**D**) the corresponding photoacoutic sigal intensity at the tumor site (*n* = 3).


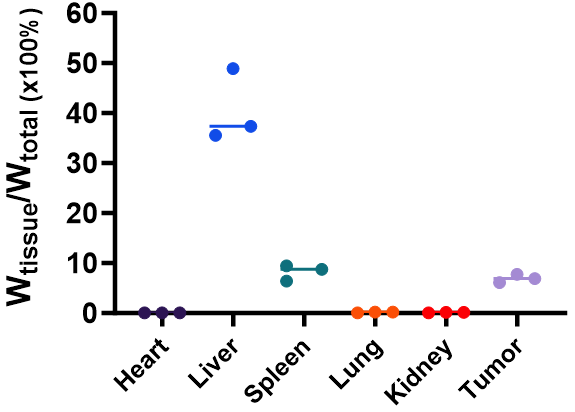


**Figure S16.** Biodistribution of AMOPs in heart, liver, spleen, lung, kidney and tumors after intravenous injection for 8h (W_tissue_ represents the mass of the Au elements in the organ tissue, and W_total_ represents the total mass of the injected Au elements) (*n* = 3).

、
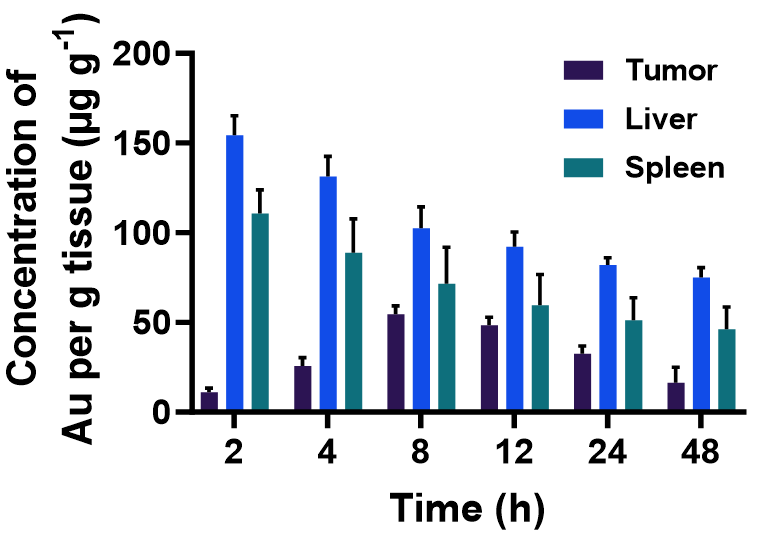


**Figure S17.** Enrichment and metabolic behavior of AMOPs (*n* = 3).


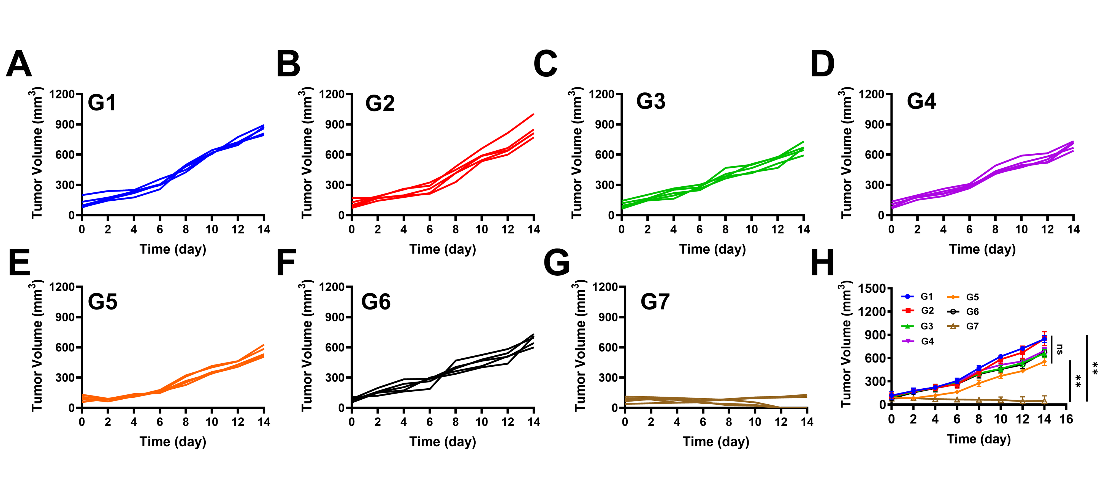


**Figure S18.** (**A-G**) Tumor growth kinetics of subcutaneous tumor assay in each group mice (*n* = 5). (**H**) Tumor volume of the mice in each group during the 14 days after treatment (*n* = 5).


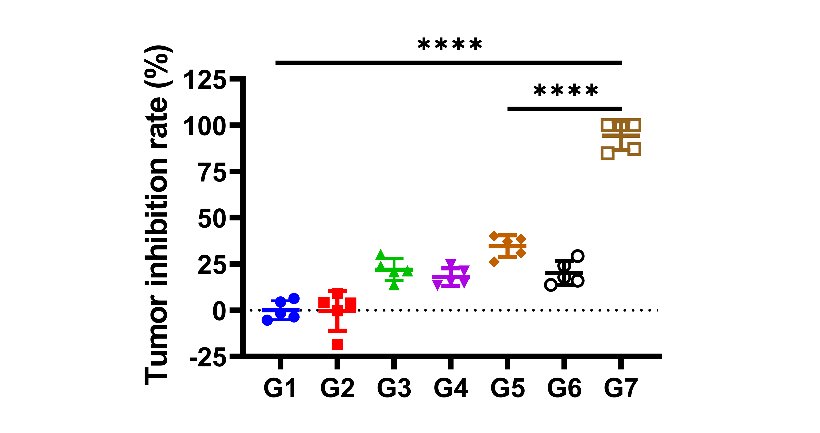


**Figure S19.** Tumor inhibition rate in each group at after treatment 14 days (*n* = 5).


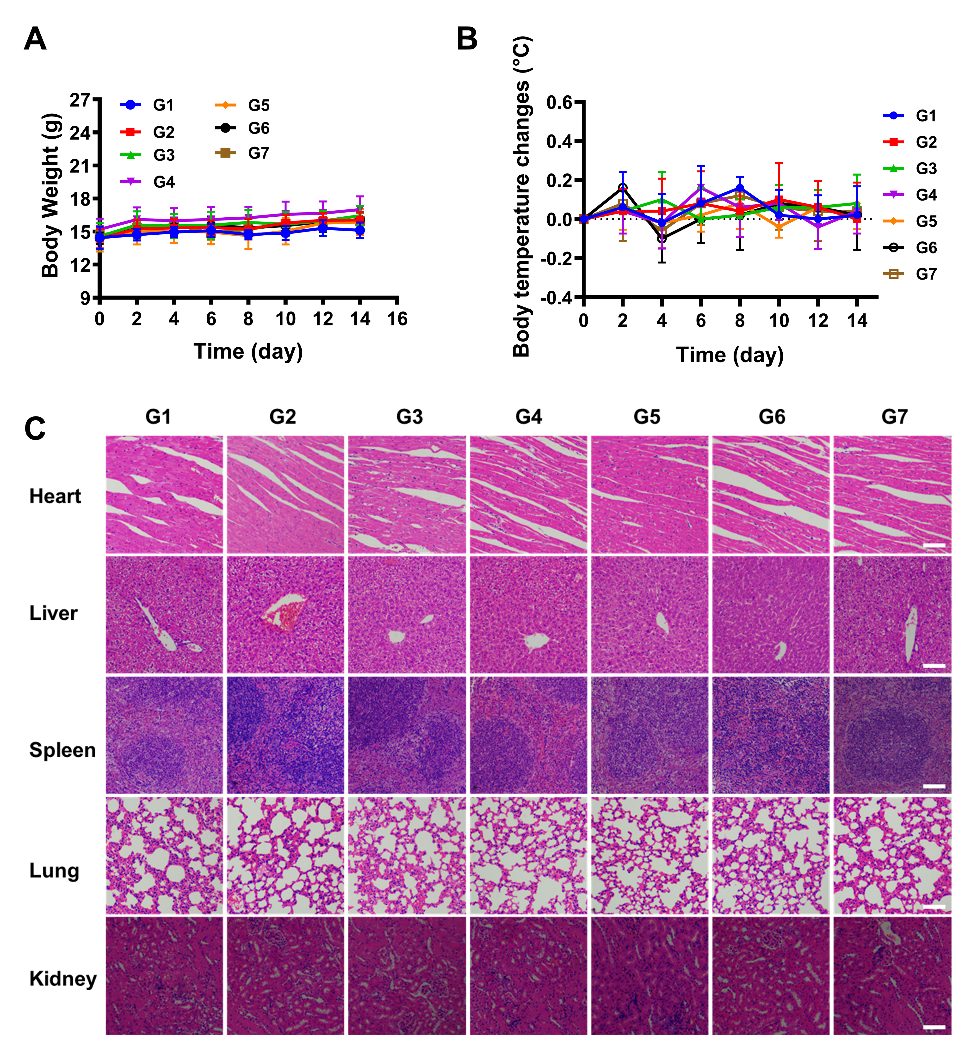


**Figure S20.** Changes of body weight (**A**) and body temperature (**B**) of the mice in each group during the 14 days after treatment (*n* = 5). (**C**) H&E staining of major organs of mice after the different treatments, scale bar: 100 µm.


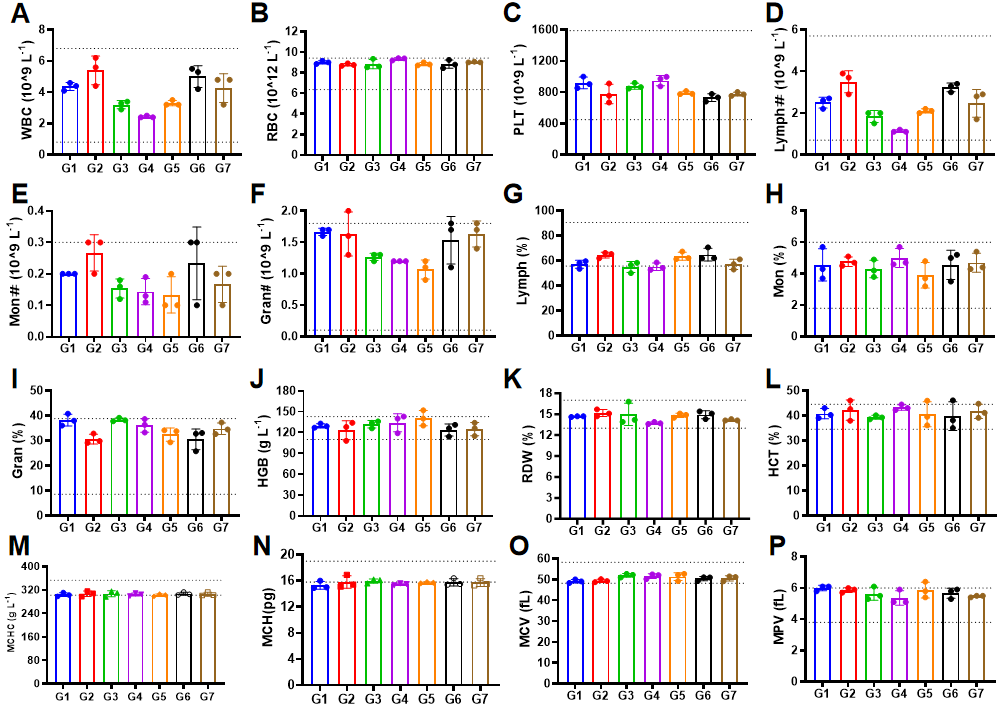


**Figure S21.** Blood routine examination of BALB/c mice with different treatments.(**A**) white blood cells (WBC). (**B**) red blood cells (RBC). (**C**) platelets (PLT). (**D**) lymphocyte (Lymph#). (**E**) monocytes (Mon#). (**F**) neutrophils (Gran#). (**G**) percentage of lymphocyte (Lymph%). (**H**) percentage of monocytes (Mon%). (**I**) percentage of neutrophils (Gran%). (**J**) hemoglobin (HGB). (**K**) red blood cell distribution width (RDW). (**L**) hematocrit (HCT). (**M**) mean corpuscular hemoglobin concentration (MCHC). (**N**) mean corpuscular hemoglobin (MCH). (**O**) mean corpuscular volume (MCV). (**P**) mean platelet volume (MPV). All of these parameters were within the normal range (black dotted line). The values are presented as the mean ± SD, (*n* = 3).


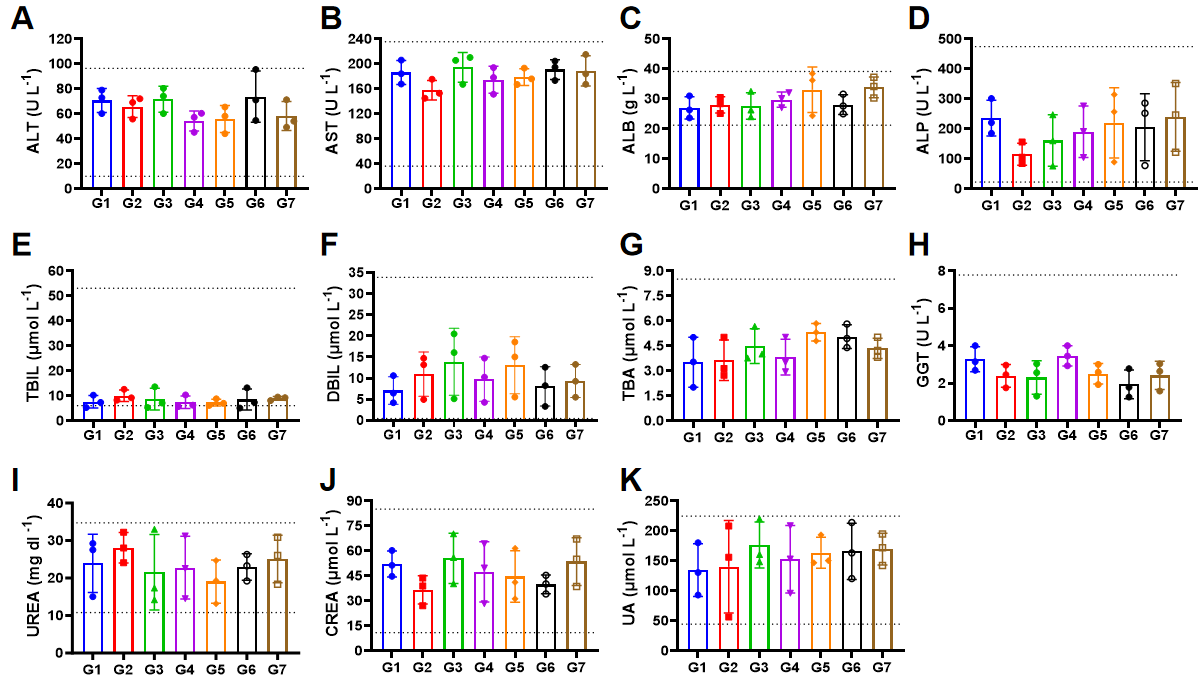


**Figure S22.** Blood biochemical tests of BALB/c mice with different treatments. (**A**) alanine amiotransferase (ALT). (**B**) aspartate aminotransferase (AST). (**C**) albumin (ALB). (**D**) alkaline phosphatase (ALP). (**E**) total bilirubin (TBIL). (**F**) direct bilirubin (DBIL). (**G**) total biliary acid (TBA). (**H**) γ-glutamyl transpeptadase (GGT). (**I**) creatinine (UREA). (**J**) creatinine (CREA). (**K**) uric acid (UA). All of these parameters were within the normal range (black dotted line). The values are presented as the mean ± SD, *n* = 3.


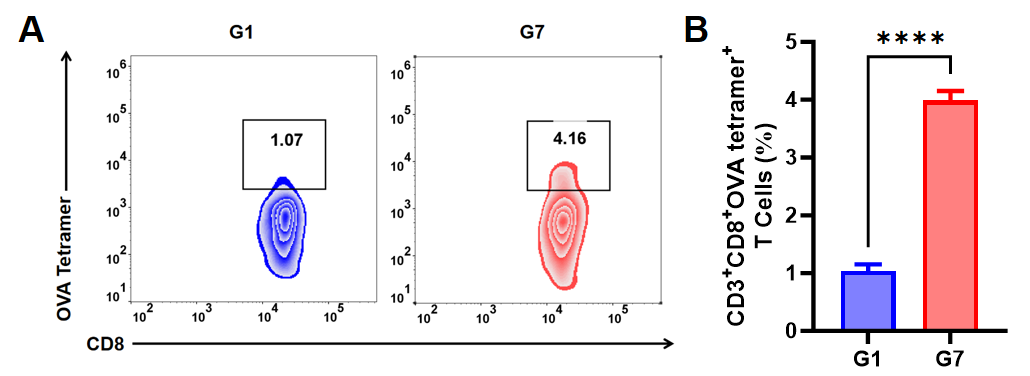


Figure S23. Characterization of antigen-specific T cell. (A) Representative flow cytometric analysis and (B) statistical data of the OVA tetramer staining of CD3^+^CD8^+^ T cells in the spleen on day 7 post-immunization (*n* = 3).


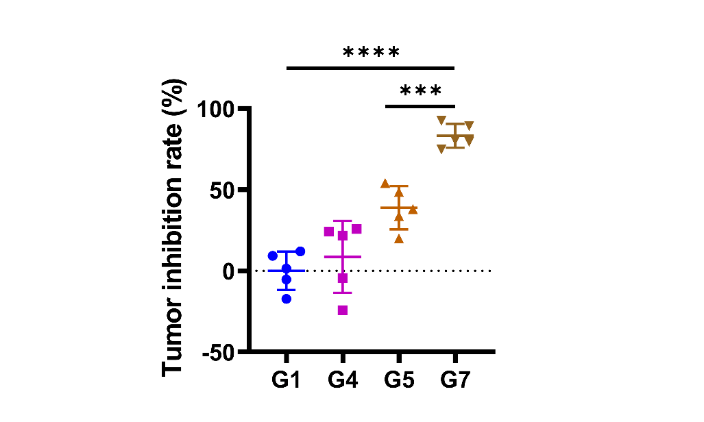


**Figure S24.** Rechallenge tumor inhibition rate of the mice in each group during the 20 days after treatment (*n* = 5).
